# Zinc finger protein ZC3H18 is abnormally expressed in esophageal cancer tissues and facilitates the proliferation of esophageal cancer cells

**DOI:** 10.3389/fimmu.2025.1556509

**Published:** 2025-02-25

**Authors:** Yujin Zhang, Yilong Wan, Jiaxi Li, Sheng Ju, Xin Tong, Ji Wu, Hao Wu, Liuqing Zhang, Shengxiang Shao, Yuhong Wang, Lingchuan Guo, Jun Zhao, Lei Cao, Dong Jiang

**Affiliations:** ^1^ Department of Thoracic Surgery, The First Affiliated Hospital of Soochow University, Suzhou, Jiangsu, China; ^2^ Institute of Thoracic Surgery, The First Affiliated Hospital of Soochow University, Suzhou, Jiangsu, China; ^3^ Department of Pathology, The First Affiliated Hospital of Soochow University, Suzhou, Jiangsu, China; ^4^ Jiangsu Institute of Clinical Immunology & Jiangsu Key Laboratory of Clinical Immunology, The First Affiliated Hospital of Soochow University, Suzhou, Jiangsu, China

**Keywords:** esophageal cancer, ZC3H18, tumor growth, cytokeratin, methylation

## Abstract

**Introduction:**

Esophageal cancer presents significant challenges due to the limited efficacy and severe side effects associated with conventional treatments. The identification of epigenetic regulatory molecules that are aberrantly expressed in tumors is crucial for elucidating the mechanisms underlying the development and progression of esophageal cancer.

**Methods:**

We performed high-throughput methylation level analysis on cancerous and adjacent tissues from 25 patients, identifying the differentially methylated gene *ZC3H18* utilizing Bismark software and data from TCGA. Esophageal cancer cell lines with ZC3H18 knockdown were used to validate the biological role of ZC3H18 in tumorigenesis *in vitro* and *in vivo*. Eukaryotic transcriptome sequencing analysis was conducted to investigate the potential mechanisms underlying ZC3H18 function.

**Results:**

We identified 30 genes exhibiting significant methylation differences between cancerous and adjacent non-cancerous tissues in 25 patients. Subsequent analysis utilizing the TCGA database revealed that the gene *ZC3H18* is aberrantly expressed in tumor tissues and is closely associated with patient prognosis. Examination of esophageal cancer tissue samples demonstrated overexpression of the ZC3H18 protein, which was positively correlated with adverse prognosis indicators, including tumor differentiation, stage, and invasion depth. ZC3H18 knockdown significantly inhibited cellular proliferation, migration, invasion, and damage repair. Additionally, ZC3H18 significantly promoted tumor growth *in vivo*. The expression of various cytokeratins was significantly reduced following the *ZC3H18* gene knockdown. ZC3H18 and multiple keratins were co-localized in esophageal cancer tissue.

**Discussion:**

*ZC3H18* gene exhibits differential methylation in esophageal cancer was positively correlated with unfavorable patient prognosis. ZC3H18 plays a critical role in the regulation of biological functions within esophageal tumors.

## Introduction

Esophageal cancer is a malignant neoplasm of the digestive system with a poor prognosis, largely attributed to its early lymphatic dissemination and the complexities of surgical treatment (1, 2). Due to the lack of effective early diagnostic methods and therapeutic targets, esophageal cancer has traditionally been treated with surgery or radiotherapy, with a 5-year overall survival rate of only 5%-10% (3).

Epigenetic abnormalities, a hallmark of nearly all human cancers, play a pivotal role in the multistep processes of tumor initiation, development, and progression (4). In esophageal cancer, epigenetic alterations—including DNA methylation, histone modifications, and loss of genomic imprinting—are critical contributors to disease pathogenesis (5). DNA methylation, one of the most well-studied epigenetic modifications, involves the transfer of a methyl group from S-adenosylmethionine to the C5 position of cytosine within CpG dinucleotides, a process catalyzed by DNA methyltransferases (6). Extensive research has identified aberrant DNA methylation as a promising biomarker for the early detection, diagnosis, and prognosis of esophageal cancer (7–10). These abnormalities are characterized by global hypomethylation and site-specific hypermethylation of CpG island promoters (11). DNA hypomethylation, in particular, has been linked to chromosomal defects and rearrangements, resulting in genomic instability. This increase in chromosomal instability is thought to significantly contribute to cancer development and progression (12, 13).

Recognizing the critical role of methylation regulation in tumorigenesis, we performed a differential methylation analysis on cancerous and adjacent tissues from 25 esophageal cancer patients. Bioinformatics techniques enable the collection and processing of large-scale transcriptomic data. By integrating bioinformatics databases and advanced methods, these techniques play a crucial role in the discovery of tumor-related biomarkers (14). Through this analysis, we identified a series of differentially methylated genes and further validated these findings using data from the TCGA public database. Among the identified genes, we focused on ZC3H18, a zinc finger protein. As no previous studies have explored the relationship between ZC3H18 and esophageal cancer, we investigated its expression, function, and potential mechanisms at both tissue and cellular levels.

## Materials and methods

### Subjects

A total of 25 patients who underwent esophagectomy at the First Affiliated Hospital of Soochow University between 2020 and 2021 were enrolled in this study. All participants were pathologically diagnosed with primary esophageal squamous cell carcinoma. Fresh cancerous and adjacent non-cancerous tissue samples were collected from each patient. The inclusion criteria were: (1) preoperative biopsy confirming esophageal cancer; (2) absence of significant tumor invasion into surrounding tissues, lymph nodes, or distant metastasis based on preoperative imaging; (3) sufficient general health to tolerate surgery; and (4) age < 80 years. The exclusion criteria included: (1) extensive adhesions within the thoracic or abdominal cavity that would complicate laparoscopic surgery and necessitate conversion to open surgery; (2) a history of prior stomach surgery preventing the use of a tubular stomach for esophageal reconstruction; (3) concurrent malignancies or severe comorbidities such as heart, lung, liver, kidney diseases, or coagulation disorders; and (4) incomplete clinical data. Detailed patient information for this cohort is provided in **Supplementary Table S1**. Additionally, a second cohort of 71 patients who underwent esophagectomy at the same institution between 2021 and 2023 was included in the study. The inclusion and exclusion criteria for this group were identical to those applied to the first cohort. Detailed patient information for the second cohort is provided in **Table 1**.

**Table 1:** Clinical and pathological features of 71 esophageal cancer patients treatment with neoadjuvant and surgery alone.

Clinical or pathological features	Neoadjuvant		P-value	Surgery alone		P-value	Total patients		P-value
	ZC3H18 expression			ZC3H18 expression			ZC3H18 expression		
	High	Low		High	Low		High	Low	
All cases	34	12		20	5		54	17	
Age			0.461			0.6232			0.2518
<70	23	10		8	3		31	13	
≥70	11	2		12	2		23	4	
Sex			0.211			0.0548			0.3312
Male	29	8		15	4		44	12	
Female	5	4		5	1		10	5	
**Tumor volume (cm3)**			**0.656**			**0.6889**			**0.7624**
<5	28	11		10	2		38	13	
≥5	6	1		10	3		16	4	
Tumor Differentiation			0.0146			0.3123			<0.0001
Well	13	29		6	3		19	32	
Poor	4	0		14	2		18	2	
Tumor stage			0.095			0.0123			0.0041
0	0	0		0	0		0	0	
I	2	4		0	2		2	6	
II	16	4		11	2		27	6	
III	14	4		9	1		23	5	
IV	2	0		0	0		2	0	
Tumor depth			0.0007			0.0568			<0.0001
T1	3	8		1	2		4	10	
T2	7	2		3	2		10	4	
T3	22	2		13	1		35	3	
T4	2	0		3	0		5	0	
LN involvement			0.2405			0.3162			0.0548
N0	14	9		7	4		21	13	
N1	11	2		8	1		19	3	
N2	8	1		4	0		12	1	
N3	1	0		1	0		2	0	
Metastasis			0.5481			0.6098			0.4209
M0	33	12		19	5		52	17	
M1	1	0		1	0		2	0	

### Tissue sequencing and bioinformatics analysis

Genomic DNA was extracted from the tissue samples of enrolled patients and subsequently treated with bisulfite to facilitate DNA methylation analysis. The bisulfite-converted DNA was then used to construct gene libraries via polymerase chain reaction (PCR). After library construction, quality control procedures were implemented, including initial quantification and insert size assessment. Once the library met the expected criteria, its effective concentration was precisely quantified using quantitative PCR (qPCR). Following quality verification, the DNA library was sequenced on the Illumina NovaSeq 6000 platform using 150-bp paired-end sequencing. Upon completion of high-throughput sequencing and acquisition of raw data, we performed data trimming to remove low-quality reads, yielding clean data for subsequent analysis. The Bismark software (BamRock, Suzhou, China) was employed for alignment against the reference genome, quality assessment of sequencing data, and identification of differentially methylated regions (DMRs). Differential methylation analysis was subsequently performed using the obtained results. To further explore the role of ZC3H18, we utilized the TCGA database along with the GEPIA tool (http://gepia.cancer-pku.cn/). For differential gene expression and survival analysis, we selected the ESCA (Esophageal carcinoma) dataset, which includes 254 patient samples.

### Cell culture

The ECA109 and KYSE150 cell lines were sourced from the American Type Culture Collection (ATCC). The ECA109 and KYSE150 cell lines were authenticated by extracting DNA using the Axygen genomic DNA extraction kit, amplifying 21-STR loci using a 21-STR amplification protocol, and detecting STR loci and the sex-determining gene Amelogenin on an ABI 3730XL genetic analyzer. These cell lines were cultured in RPMI-1640 medium (Bio-Ind, Beit HaEmek, Israel) supplemented with 10% fetal bovine serum (FBS) and 1% penicillin-streptomycin (Beyotime, Shanghai, China, #C0222). Cultures were maintained in a humidified incubator at 37°C with 5% CO2.

### Small interfering RNA-mediated knockdown of ZC3H18

Small interfering RNAs (siRNAs) targeting ZC3H18 were purchased from Suzhou Hongxun Biotechnology Co., Ltd. (Suzhou, China), with an empty vector serving as the control (si-nc). ZC3H18 siRNA sequences were GGGGTGAGGGCTTCTGATCT and TCGTCGGAGTGATTATCTTCCT. These siRNAs were introduced into KYSE150 and ECA109 cells using DharmaFect1 (Suzhou Hongxun Biotechnology Co., Ltd., Suzhou, China). KYSE150 and ECA109 cells were seeded into 6-well plates at a density of 1×10^5 cells per well. Forty-eight hours post-transfection, the knockdown efficiency was evaluated through Western blot analysis.

### Western blot

Cells were washed with ice-cold PBS and lysed using ice-cold lysis buffer composed of 0.1 M Tris (pH 6.8), 4% sodium dodecyl sulfate (SDS), 20% glycerol, and 0.2 M dithiothreitol (DTT). Protein concentration was quantified using an enhanced BCA protein assay kit (Beyotime, #P0010). For Western blot analysis, 30 μg of total protein was separated by 10% SDS-PAGE (Beyotime, #P0012AC) and subsequently transferred to 0.45 μm polyvinylidene fluoride (PVDF) membranes (GE Healthcare Life Sciences, Germany). Membranes were blocked with 5% bovine serum albumin (BSA; Fcmacs, Nanjing, China, #FMS-WB021) for 1 hour, then incubated overnight at 4°C with the specified primary antibody targeting ZC3H18 (1:1000 dilution, Cat No: 66481-1-Ig, Proteintech, China). The following day, membranes were incubated at room temperature for 1 hour with horseradish peroxidase (HRP)-conjugated secondary antibodies (Beyotime, Shanghai, China). Protein bands were visualized using an electrochemiluminescence (ECL) reagent (NCM Biotech, Suzhou, China, #10100) and detected with the ChemiDoc™ MP Imaging System (Bio-Rad).

### Cell proliferation assay

Tumor cell proliferation was assessed using the CCK-8 colorimetric assay (NCM Biotech, Suzhou, China) according to the manufacturer’s protocol. Given the differential growth rates of the two cell lines, 3000 ECA109 cells and 2000 KYSE150 cells were seeded per well in 96-well plates, each containing 100 μL of cell culture medium. After a 48-hour incubation period, the cells were maintained in complete medium. One hour prior to measurement, the medium was replaced with serum-free medium containing 10 μL of the CCK-8 reagent per well. Cells were then incubated at 37°C for 1 hour, and absorbance was measured at 450 nm. Cell proliferation was monitored every 24 hours for a total of five cycles.

### Colony formation assay

Clonogenic assays were conducted in 6-well plates to assess the proliferative capacity of ECA109 and KYSE150 cells. A total of 1000 ECA109 cells and 800 KYSE150 cells were seeded per well and cultured in complete medium for 7 days. Following this incubation period, the cells were fixed with 4% methanol at room temperature for 20 minutes and then stained with crystal violet for an additional 20 minutes. After staining, the plates were gently washed with tap water to remove excess dye. Colonies containing at least 50 cells were counted to evaluate cell proliferation.

### Apoptosis assay

Cell apoptosis was evaluated using the Annexin V-PE/7-AAD double-staining apoptosis detection kit (Vazyme Biotech Co., Nanjing, China, #A213-01). After transient transfection with siRNA, cells were seeded into 6 cm dishes and cultured to confluence, with three replicates per condition. Annexin V-PE was used to identify early apoptotic cells, while 7-AAD was employed to detect late apoptosis. Cells were washed with cold phosphate-buffered saline (PBS) and resuspended in 100 μL of binding buffer. The cells were then incubated in the dark with 5 μL of APC-Annexin V and 5 μL of 7-AAD solution for 10 minutes. Apoptosis was analyzed using a flow cytometer (Beckman Coulter, CA, USA).

### Transwell migration and invasion assays

Transwell assays were conducted to assess the migration and invasion abilities of KYSE150 and ECA109 cells. For invasion assays, the upper chamber (8 µm pore size) was coated with 20 µL of Matrigel (diluted 1:4) and incubated at 37°C for 30 minutes to allow gel formation. Both cell lines were seeded in serum-free medium at a density of 40,000 cells per well, while the lower chamber was filled with 600 μL of medium containing 20% fetal bovine serum (FBS) to promote cell migration and invasion. For ECA109 cells, migration assays were fixed and stained after 48 hours, while invasion assays were fixed and stained after 60 hours. For KYSE150 cells, both migration and invasion assays were fixed and stained after 48 hours. After incubation, cells were fixed with 4% paraformaldehyde for 15 minutes and stained with crystal violet (Beyotime, #C0121) for 15 minutes. Cells that had migrated or invaded to the lower surface of the membrane were photographed and counted using an inverted microscope. Five random fields per group were counted at 100x magnification, and the number of invasive cells was recorded for each group in triplicate.

### Construction of stable cell lines with ZC3H18 knockdown and overexpression

Stable knockdown and overexpression cell lines of ZC3H18 in KYSE150 and ECA109 were established through lentiviral-mediated gene manipulation, with an empty vector serving as a control. ShRNA sequences targeting ZC3H18 were specifically designed to ensure both high specificity and efficiency. The shRNA sequences were as follows: sh1: GGAATGAATTGTAGGTTTATA, sh2: GGCCGGTAGTTGATGAAATTT, and sh3: GCCTTACGCAGACCCTTATTA. These sequences were cloned into a lentiviral expression vector containing a puromycin resistance gene, facilitating selection and stable integration of the shRNA into the KYSE150 cell genome. For ECA109 cells, the sh1 sequence (GGAATGAATTGTAGGTTTATA) was specifically cloned for integration. The lentiviral vectors were validated by enzymatic digestion and sequencing to confirm correct insertion of the shRNA sequences. Lentiviral particles were produced by co-transfecting the shRNA-containing vector, along with packaging and envelope plasmids, into 293T cells using a calcium phosphate transfection method. The viral supernatant was harvested, filtered, and concentrated to achieve a high titer of lentiviral particles. Target cells were seeded at 1×10^5 cells per well in 24-well plates and incubated overnight to allow for cell attachment. The next day, the culture medium was replaced with fresh medium containing the concentrated lentiviral particles and polybrene (6-8 μg/mL) to enhance viral transduction. Cells were incubated for 4-6 hours at 37°C, after which the medium was replaced with complete growth medium, and incubation continued for an additional 72 hours. Following transduction, puromycin was added to the culture medium at a final concentration of 2-3 μg/mL for selection of transduced cells. Selection was maintained for 2-3 days, after which puromycin-resistant colonies were isolated, expanded, and further characterized. For the ZC3H18 overexpression KYSE150 cell line, we coding sequence of human ZC3H18 (NM_001294340.2) was cloned into the lentiviral vector pLV-CMV-FLAG using EcoRI and XhoI sites. HEK293T cells were used for lentiviral packaging. After infection, KYSE150 cells were selected with 2 μg/mL puromycin for 7 days. Overexpression of ZC3H18 was confirmed by qPCR, showing a 10-fold increase in mRNA levels, and by Western blot, revealing a FLAG-tagged protein band at the expected molecular weight.

### Establishment of mouse xenograft model

Female BALB/c nude mice (4-5 weeks old) were obtained from Shanghai SLAC Laboratory Animal Co., Ltd. All mice were subcutaneously injected with KYSE150 and ECA109 cells (2.5×10^7 cells/mL, 100 µL per injection). Following cell inoculation, the mice were randomly assigned to five experimental groups: KYSE150 control, KYSE150 knockdown, KYSE150 overexpression, ECA109 control, and ECA109 knockdown. After 8 days of treatment, the mice were euthanized by decapitation, and tumor tissues were excised and measured. Tumor volume (mm³) was calculated using the formula: (length × width²)/2. All data from the animal experiments were collected in a blinded manner to ensure unbiased analysis. These experiments were conducted in accordance with the guidelines approved by the Animal Care and Treatment Committee of the National Key Laboratory of Biotherapy, Soochow University (Approval No: 2019121).

### Eukaryotic transcriptome sequencing analysis

The *ZC3H18* gene knockdown KYSE150 and ECA109 cells, mediated by siRNA, along with their respective control cell lines, were sent to CapitalBio Technology Co., Ltd. (Beijing, China) for further assays and analysis.

### Multiplex immunofluorescence

Multiplex immunofluorescence was performed on esophageal cancer tissue sections using specific antibodies targeting ZC3H18, KRT6A, and KRT14. Briefly, tissue slides were deparaffinized with xylene and rehydrated through a graded ethanol series. Antigen retrieval was performed by boiling the slides in sodium citrate buffer (pH 6.0) for 15 minutes, followed by blocking endogenous peroxidase activity with 3% hydrogen peroxide at room temperature for 15 minutes. Non-specific binding was prevented by incubation with a goat serum solution for 30 minutes. The slides were then incubated overnight at 4°C with the primary antibody against ZC3H18 (1:500, Cat No: 66481-1-Ig, Proteintech, China). After washing, the slides were incubated at room temperature for 30 minutes with horseradish peroxidase (HRP)-conjugated secondary antibody. Subsequently, Tyramide Signal Amplification (TSA) Fluorescein (Cat No: NEL701A001KT, Akoya) was applied for 10 minutes at room temperature to amplify the signal. Following each staining cycle, slides were heated in a microwave to dissociate the Ab-TSA complex and blocked with goat serum to prevent non-specific binding. The process was repeated for KRT6A (1:400, Cat No: 10590-1-AP, Proteintech, China) with TSA Cyanine 3 (Cat No: NEL704A001KT, Akoya) labeling, and for KRT14 (1:200, Cat No: 10143-1-AP, Proteintech, China) with TSA Cyanine 5 (Cat No: NEL705A001KT, Akoya) in the final staining run. For nuclear visualization, DAPI (DAPI Fluoromount-G, SouthernBiotech, USA) was applied, and slides were mounted for imaging. Representative images were captured and analyzed using the Mantra and inForm software platforms (Akoya Biosciences).

### Statistical analysis

Statistical analyses were performed using GraphPad Prism 10.0 and SPSS software. Overall survival (OS) was defined as the time from the start of treatment to the date of death. It should be emphasized that OS was recorded in only 25 patients. The chi-square test was used to analyze the correlation between categorical variables. Correlations between variables were analyzed using Spearman rank correlation analysis. Survival analysis was performed using Kaplan-Meier curves, and Cox proportional risk models were used for univariate and multivariate analyses p<0.05 was defined as statistically significant difference.

## Results

### Exploration and screening of differential methylation biomarkers between esophageal cancer and adjacent non-cancerous tissues

**Figure 1:**
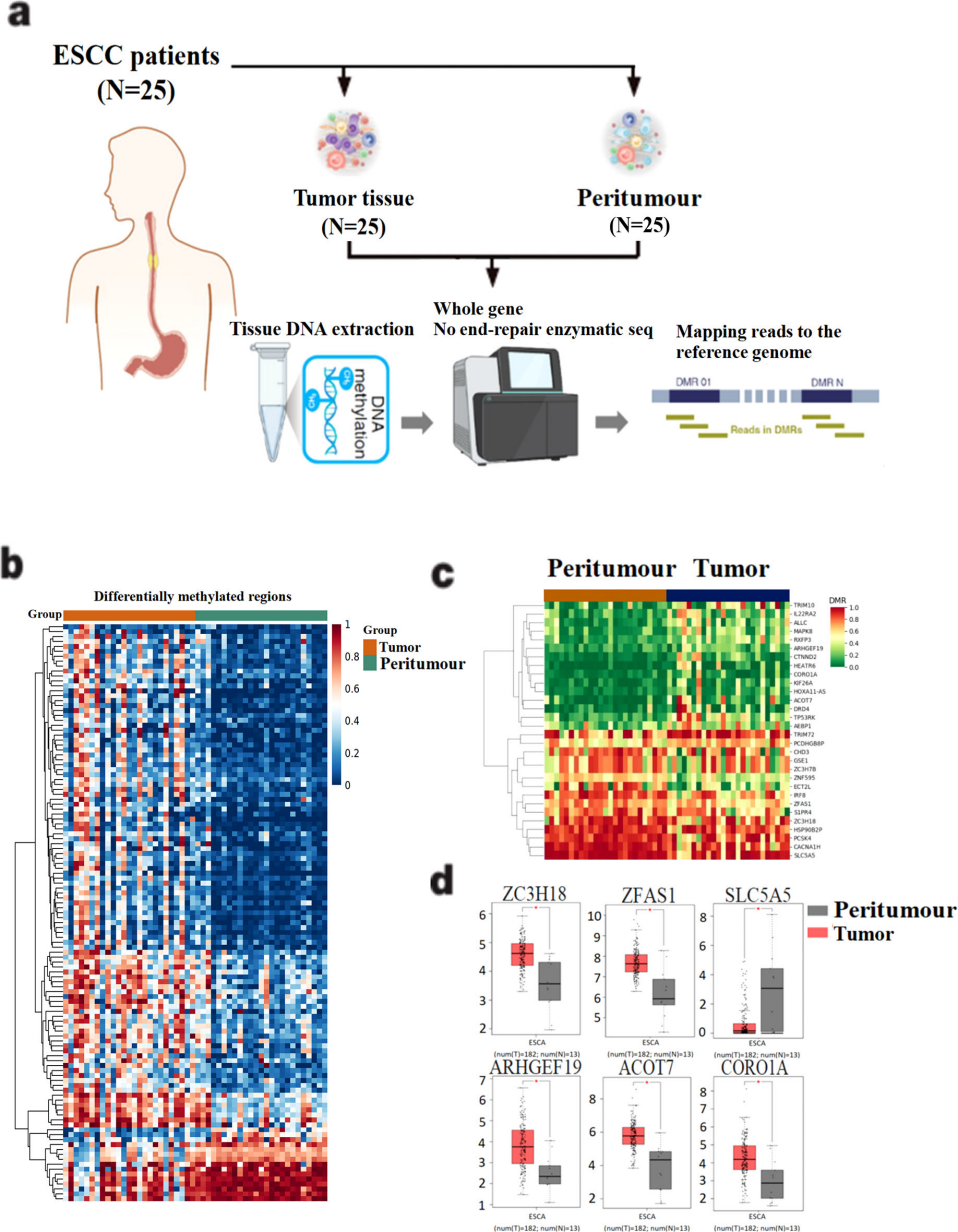
Screening of differentially methylated genes in 25 esophageal cancer patients. **(A)** Methylation sequencing workflow diagram of 25 esophageal cancer patients. DNA was extracted from tissue samples and DNA bisulfite was treated to construct gene library. After the gene library is constructed, the quality of the library is tested and sequenced. **(B)** Differential methylation data were obtained after mapping and sequencing quality assessment. **(C)** 30 differentially methylated genes were identified by setting filtering conditions. **(D)** Gene mRNA expression differences between tumor and peritumour were analyzed in the TCGA esophageal cancer dataset. The horizontal axis is Expression−log_2_(TPM+1), and the vertical axis is the ESCA dataset.

We first performed methylation sequencing on 25 paired clinical esophageal cancer tissue samples and visualized the results for analysis (**Figure 1A**, **Supplementary Table S1**). The methylation sequencing revealed variability in methylation levels across tumor tissue samples, while normal tissue samples exhibited more consistent methylation patterns (**Figure 1B**).

To identify disease-related differentially methylated genes, we applied the following four screening criteria: (1) Q value < 0.05, (2) ∣meth._diff∣ > 10, (3) annotation located in the promoter region, and (4) ∣distance to TSS∣ < 2000 bp. Using these criteria, we identified 30 differentially methylated candidate genes, of which 13 were hypomethylated and 17 were hypermethylated in tumor tissues (**Figure 1C**). To further understand the functional implications, we analyzed the mRNA expression levels of these 30 differentially methylated genes using the TCGA esophageal cancer dataset. Six genes—*ARHGEF19, CORO1A, ACOT7, ZC3H18, SLC5A5*, and ZFAS1—showed statistically significant differential expression between tumor and normal tissues (P < 0.05) (**Figure 1D**).

### Abnormal overexpression of methylation-regulated ZC3H18 gene in esophageal cancer tissues

**Figure 2:**
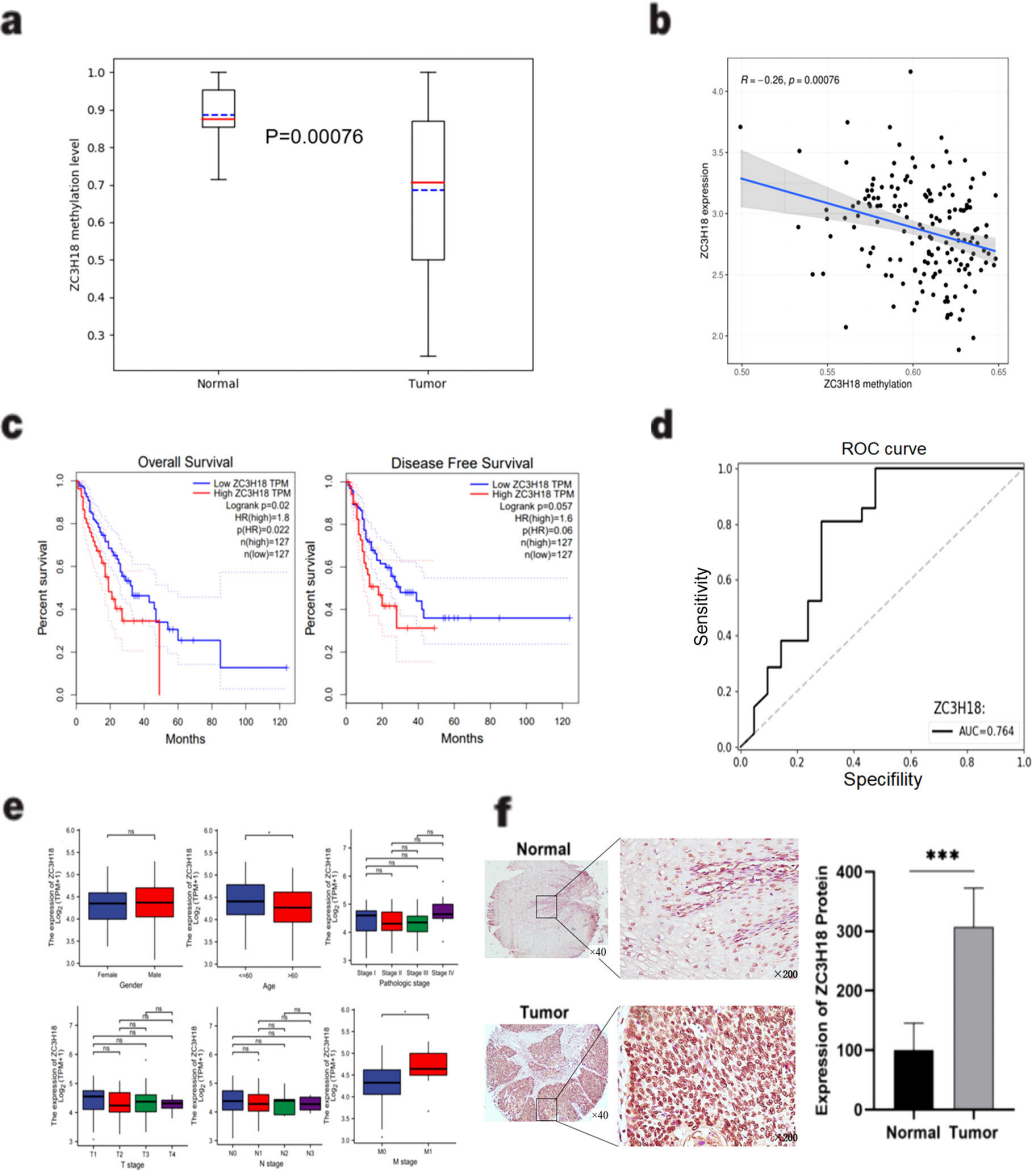
Identification and validation of the ZC3H18 gene. **(A)** Comparison of the methylation levels of ZC3H18 between esophageal cancer and adjacent normal tissues based on the methylation data of the ZC3H18 gene in the gene library we constructed in **Figure 1**. Used the “pandas” package and the “sklearn” package in Python language to visualize the results. **(B)** A significant negative correlation between ZC3H18 expression and methylation level based on the TCGA database. The results were analyzed and visualized using the “limma” package in R language. **(C)** Prognostic analysis of ZC3H18 in esophageal cancer patients based on the TCGA esophageal cancer dataset on the GEPIA online website. **(D)** The diagnostic value of ZC3H18 based on its methylation data of our gene library. The results were visualized using the “pandas” and “sklearn” packages in Python. **(E)** Based on the TCGA database, the correlation between ZC3H18 expression and clinicopathological characteristics was analyzed. * represents P < 0.05. **(F)** Immunohistochemical staining and quantitative analysis were performed on esophageal cancer and normal tissues. *** represents P < 0.001. The scale bar in the left image is 400 µm, and the scale bar in the right image is 100 µm. ns, non-specific.

Through a comparative analysis of gene methylation levels and expression between cancerous and adjacent non-cancerous tissues, we identified the zinc finger protein ZC3H18 as a candidate of interest. Bioinformatics analysis of results from the differential gene methylation analysis of 25 patients confirmed that *ZC3H18* exhibited significantly lower methylation levels in esophageal cancer tissues (P = 0.0075) (**Figure 2A**). Further analysis revealed a significant negative correlation between *ZC3H18* expression and its methylation status in esophageal cancer tissues (R = -0.26, P = 0.00076) (**Figure 2B**).

Prognostic analysis indicated that esophageal cancer patients with high ZC3H18 expression had significantly reduced overall survival (OS) (P = 0.02), while a reduction in disease-free survival (DFS) was observed but did not reach statistical significance (P = 0.057) (**Figure 2C**). Receiver operating characteristic (ROC) curve analysis yielded an area under the curve (AUC) of 0.764 for *ZC3H18*, highlighting its potential as a diagnostic biomarker for esophageal cancer (**Figure 2D**). Furthermore, TCGA database analysis revealed that *ZC3H18* expression was significantly associated with patient age and M stage (P < 0.05) (**Figure 2E**).

To evaluate the protein expression of ZC3H18 in esophageal cancer tissues, we performed immunohistochemical staining on tissue sections from patients who underwent surgical resection alone and those who received neoadjuvant immunotherapy. Localization analysis revealed that the ZC3H18 protein was predominantly localized within the nuclei of tumor cells in both cancerous and adjacent non-cancerous tissues (**Figure 2F**). Quantitative analysis showed a significantly higher number of ZC3H18-positive cells in esophageal cancer tissues compared to adjacent non-cancerous tissues (P < 0.001) (**Figure 2F**). We further investigated the relationship between ZC3H18 expression and the pathological parameters of the patients. In the surgery-alone group, ZC3H18 expression was significantly correlated with tumor stage (P = 0.0123). In the neoadjuvant therapy group, ZC3H18 expression was significantly associated with tumor differentiation (P = 0.0146) and tumor depth (P = 0.0007). When combining data from both groups, ZC3H18 expression was significantly correlated with tumor differentiation (P < 0.0001), tumor depth (P < 0.0001), and tumor stage (P = 0.0041). However, no significant correlations were found with other clinical features, such as age, sex, tumor volume, lymph node (LN) involvement, or metastasis (**Table 1**).

### Knockdown of ZC3H18 gene inhibits tumor biological functions of esophageal cancer cells *in vitro* and *in vivo*

**Figure 3:**
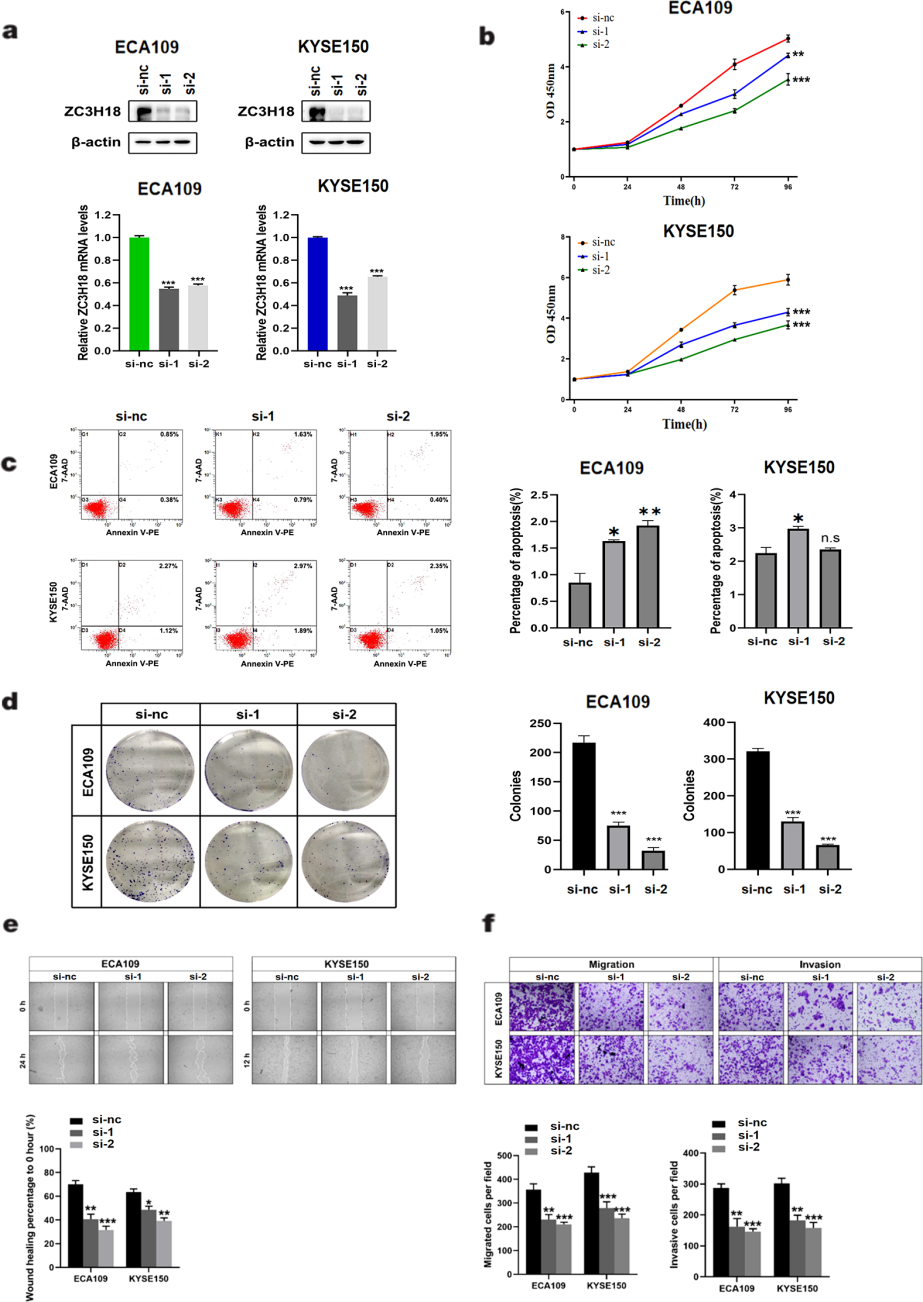
Knockdown of ZC3H18 inhibits the *in vitro* proliferation, migration, and invasion of two esophageal cancer cell lines. **(A)** Western blot analysis of ZC3H18 in two siRNA-treated esophageal cancer cell lines. β-actin was used as the loading control. **(B, D)** Cell viability was assessed in two cell lines using CCK-8 and colony formation assays, respectively. **(C)** Cell apoptosis was detected by flow cytometry. **(E)** Wound-healing ability was evaluated by scratch assay. **(F)** Migration and invasion abilities of the two cell lines were evaluated by transwell assays.

Given the high expression of ZC3H18 in tumor tissues and its significant negative correlation with patient prognosis, we sought to investigate whether knockdown of ZC3H18 could inhibit tumor growth. Using siRNA, we successfully achieved effective knockdown of ZC3H18 in two esophageal cancer cell lines, ECA109 and KYSE150 (**Figure 3A**). Knockdown of ZC3H18 resulted in a marked reduction in both cell proliferation and colony-forming ability (**Figures 3B, D**). To further assess the functional consequences of ZC3H18 knockdown, we evaluated apoptosis and observed a significant increase in cell apoptosis following the loss of ZC3H18 function (**Figure 3C**). Additionally, transwell and scratch assays revealed a significant suppression of cell migration, invasion, and the ability to repair cellular damage in ZC3H18-knockdown cells (**Figures 3E, F**).

**Figure 4:**
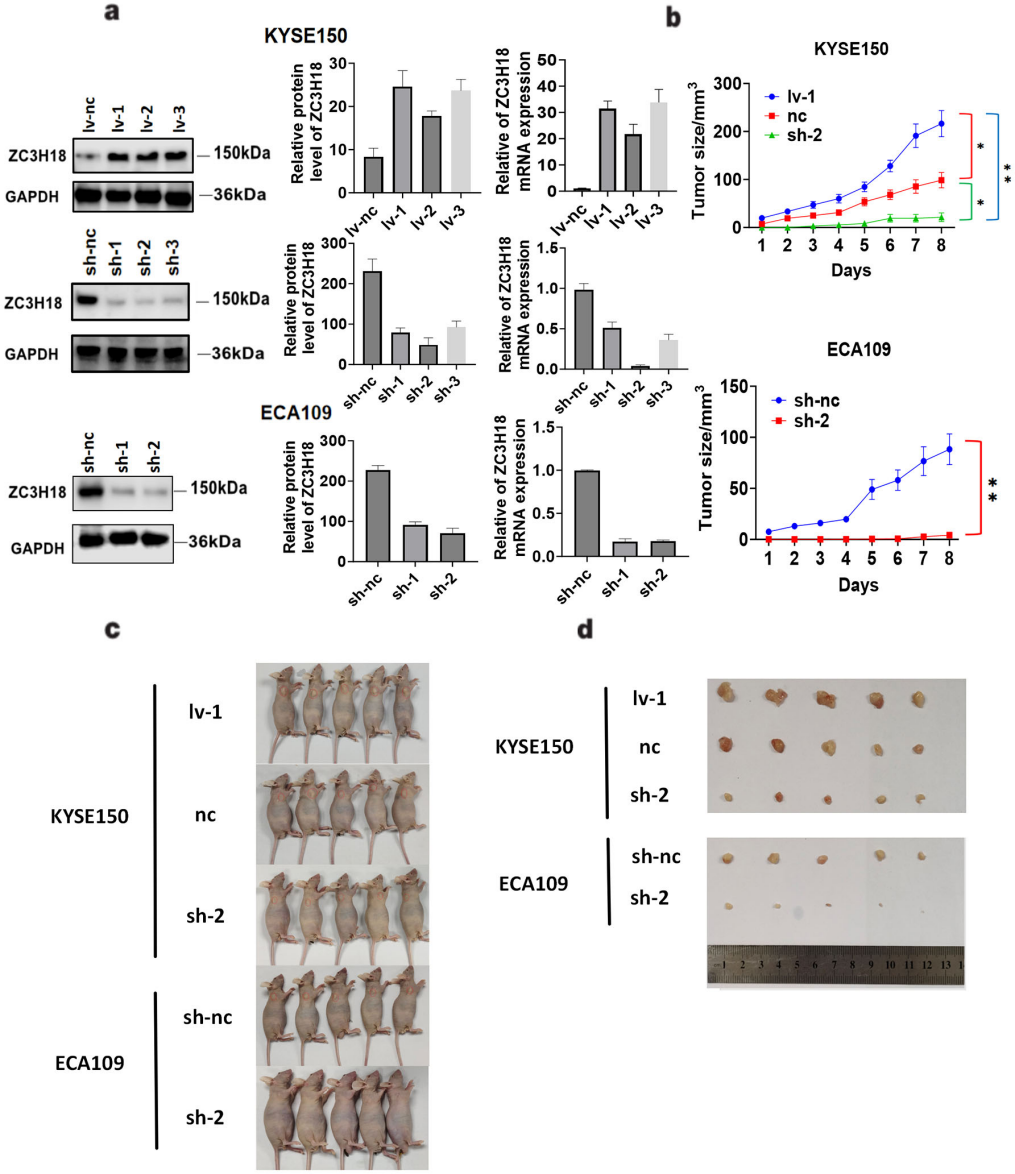
Tumorigenic effects of stable knockdown and overexpression of ZC3H18 in two esophageal cancer cell lines *in vivo*. **(A)** Validation analysis of ZC3H18 gene knockdown and overexpression of the esophageal cancer cell lines KYSE150 and ZC3H18 gene knockdown of ECA109. Lv 1,2,3 – lentivirus ZC3H18 overexpression 1,2,3. sh 1,2,3 – short hairpin ZC3H18 knockdown 1,2,3. nc – nonspecific negative control. **(B)** Tumor volume changes in mice, data expressed as mean ± SEM. **(C, D)** Representative images of tumors harvested after 8 days in ZC3H18 knockdown and overexpression xenografts.

We generated stable ZC3H18 knockdown and overexpression cell lines in KYSE150 and ECA109 esophageal cancer cell lines (**Figure 4A**) and investigated the impact of ZC3H18 modulation on tumor formation *in vivo*. The results demonstrated that ZC3H18 knockdown significantly inhibited tumor growth, while ZC3H18 overexpression markedly promoted tumor proliferation (**Figures 4B–D**).

### ZC3H18 regulates multiple cytokeratins expressions and co-localizes with cytokeratin in tumor cells of clinical samples

**Figure 5:**
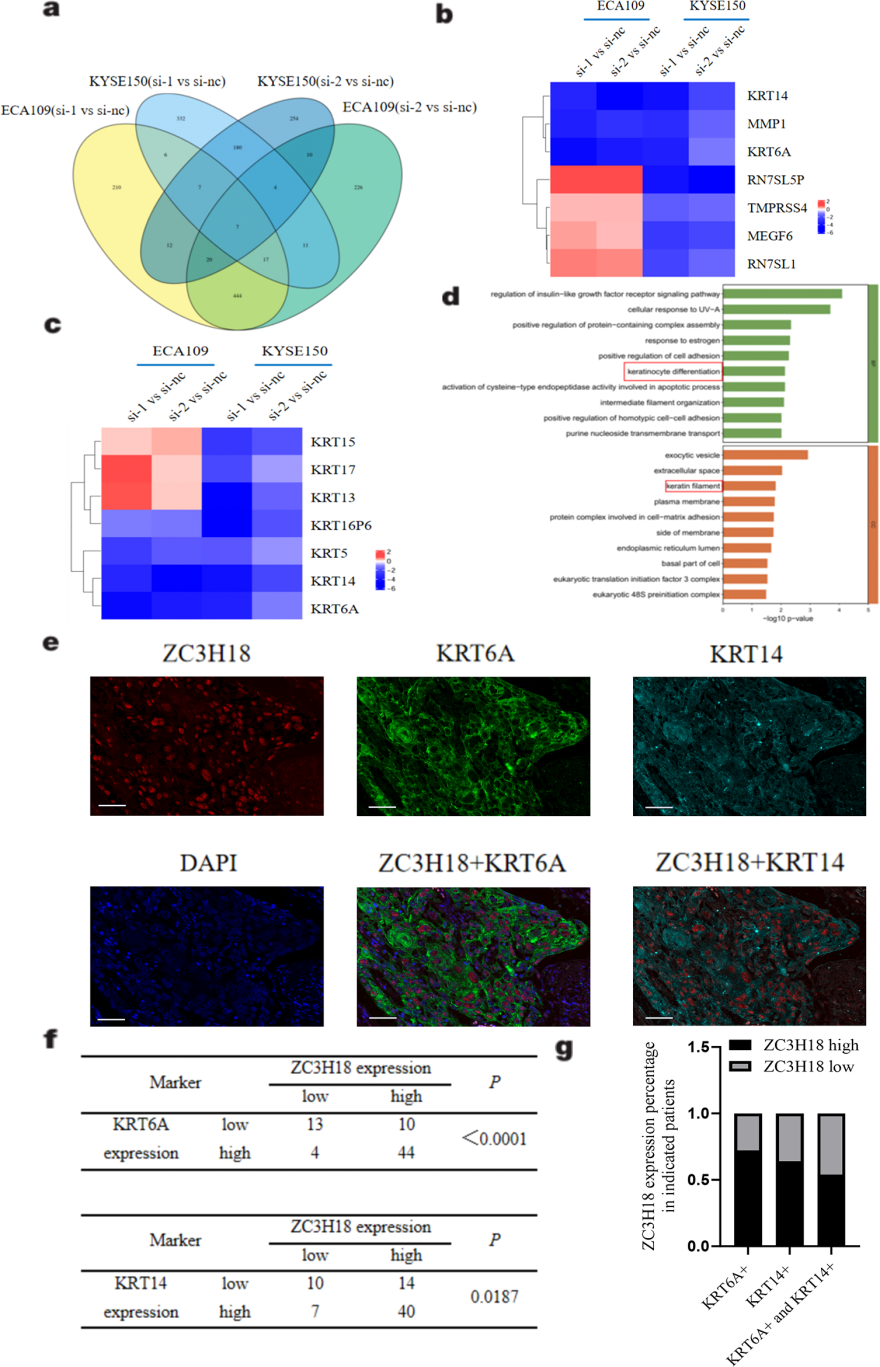
Exploring the regulatory mechanism of ZC3H18 on tumors through eukaryotic transcriptome sequencing. **(A)** Venn diagram of differentially expressed genes between two knockdown sites and the control in two cell lines. si 1,2 – small interfering ZC3H18 knockdown 1,2. nc – nonspecific negative control. **(B)** Heatmap of the expression of seven genes with significant differences across the four compared groups. **(C)** Heatmap of the expression of seven cytokeratin genes across the four compared groups. **(D)** Pathway enrichment analysis of 53 genes with differential expression in at least three groups from the Venn diagram, showing BP and CC enriched pathways. **(E)** Multicolor immunofluorescence staining of ZC3H18, KRT6A, and KRT14 in tissue chips of 71 esophageal cancer patients. Scale bar: 50um. **(F, G)** Statistical analysis of correlation and co-localization of ZC3H18 with KRT6A and KRT14.

To investigate the potential regulatory mechanisms underlying *ZC3H18* gene function, we performed RNA sequencing analysis on cells with ZC3H18 knockdown. Differentially expressed genes between the experimental and control groups were identified and summarized. A Venn diagram was constructed, revealing seven common differentially expressed genes: MEGF6, RN7SL1, MMP1, RN7SL5P, KRT6A, KRT14, and TMPRSS4 (**Figures 5A, B**). Notably, several of these genes belong to the keratin family, prompting us to further explore their roles in the development of esophageal squamous cell carcinoma. Our analysis showed consistent downregulation of four keratins (KRT5, KRT14, KRT6A, and KRT16P6) across all four experimental groups (**Figure 5C**). We then performed pathway enrichment analysis on 53 genes exhibiting significant differential expression across at least three of the four groups. This analysis revealed substantial alterations in multiple signaling pathways, particularly those associated with keratinocyte differentiation and keratin filament formation (**Figure 5D**).

To further explore the relationship between ZC3H18 and cytokeratins, we conducted multicolor immunofluorescence staining on tissue microarrays derived from esophageal cancer patients. The results clearly demonstrated co-expression of ZC3H18 with cytokeratins, particularly KRT6A and KRT14 (**Figures 5E, F**). Quantitative analysis revealed that 84% of ZC3H18-high expressing cells were positive for KRT6A, while 72% were positive for KRT14. Moreover, 54% of ZC3H18-high expressing cells were co-positive for both KRT6A and KRT14 (**Figures 5F, G**).

## Discussion

The protein encoded by the *ZC3H18* gene is primarily localized to nuclear speckles, where it functions as a component of a ribonucleoprotein complex involved in RNA destabilization. Its critical functional involvements include mediating mRNA cap-binding complex activity and facilitating protein-macromolecule interactions (15). Furthermore, previous studies have demonstrated that ZC3H18 interacts with E2F binding sites in the BRCA1 promoter, thereby promoting the recruitment of E2F4 to adjacent E2F sites and enhancing BRCA1 transcription (16). These findings suggest that ZC3H18 not only plays a critical role in RNA destabilization but also serves as a transcriptional cofactor, contributing to the regulation of gene expression.

This study screened and found significant differences in the methylation levels of series of genes between cancerous and adjacent tissues in clinical samples. By setting four criteria, we narrowed down the target genes and identified 30 genes with significant differential methylation. As seen in the figures, methylation changes are relatively consistent in adjacent tissues, while cancer tissues exhibit greater variation, reflecting heterogeneity in methylation levels across different patients. We further compared these results with the TCGA database and identified six genes. Among them, ACOT7, SLC5A5, ARHGEF19, and CORO1A exhibited consistent trends in expression and methylation differences between cancerous and adjacent tissues, while ZC3H18 and ZFAS1 displayed opposite trends. Considering that gene expression is often inhibited by methylation regulation, we focused our research on these two genes. Since ZFAS1 has already been studied in esophageal cancer, such as exosomal ZFAS1 promoting proliferation, migration, and invasion of esophageal squamous cell carcinoma by upregulating STAT3 and downregulating miR-124 while inhibiting apoptosis (17), we chose ZC3H18 for further investigation.

The application of bioinformatics approaches has opened new directions for identifying diagnostic markers for various malignant tumors. This includes cancers such as prostate cancer and bladder cancer, offering innovative insights into tumor biology and potential therapeutic targets (18, 19). Our results showed that in the absence of ZC3H18, most cytokeratin genes were downregulated. Studies have shown that keratin intermediate filaments are the main cytoskeletal proteins that maintain epithelial integrity. Mutations that disrupt the keratin cytoskeleton or post-translational modifications that cause reorganization make epithelial cells more susceptible to tissue damage and various stresses, while the loss of keratin expression is a hallmark of epithelial-mesenchymal transition (20, 21). Additionally, keratins play roles in determining cell size, regulating signal transduction, translation, expression, cell proliferation, organelle transport, malignant transformation, and responses to various stresses (22). Modified cytokeratins accumulate in various cancers, and this modification enhances cancer invasiveness by disrupting the cytoplasmic cytokeratin network and allowing free movement. The phenotype of cancer cells can change, promoting invasion and metastasis. Modified cytokeratins also deregulate mitosis and apoptosis, leading to immortalization (23). Therefore, ZC3H18 may regulate the proliferation and invasion of squamous epithelial cancer cells through keratin regulation, which aligns with our experimental results (**Figure 3**). Cytokeratins are closely associated with esophageal squamous cell carcinoma. For example, clinical pathological analysis has shown that cytokeratin is co-expressed with tumor cells in esophageal spindle cell carcinoma, and cytokeratin could serve as a novel tumor marker (24). KRT19 mRNA levels could be used as an indicator for prognosis evaluation and treatment selection in esophageal cancer patients (25). The reduced expression of KRT15 in esophageal squamous cell carcinoma tissues and patient serum suggests its important role in the development of esophageal squamous cell carcinoma (26). CD24 and CK4 could be predictive biomarkers for the effectiveness of chemoradiotherapy in esophageal cancer (27). KRT6A is a type I keratin. During keratinocyte differentiation, the main roles of KRT6A include its rapid upregulation in response to skin injury or stimulation, providing structural support to keratinocytes and promoting barrier repair (28). Additionally, KRT6A influences related signaling pathways, such as MAPK, to prevent premature keratinocyte differentiation and maintain epidermal barrier function (29). According to relevant studies, KRT6A interacts with KRT16 or KRT17 to form mechanically stable keratin filament networks, enhancing the tensile strength of cells in stressful environments (30). KRT14 is a type II keratin and a critical marker of basal keratinocytes. Its main roles in keratinocyte differentiation and keratin filament formation include its high expression in the basal layer of the epidermis, where it forms the cytoskeleton in conjunction with KRT5, providing mechanical strength to basal cells (31). Moreover, KRT14 regulates the differentiation process of keratinocytes by interacting with signaling molecules (such as 14-3-3 proteins), thereby influencing the formation of the epidermal barrier (32). These findings suggest that ZC3H18 may regulate the pathogenesis and progression of esophageal cancer through the modulation of these keratin-related pathways, underscoring its potential role in tumorigenesis and tumor progression.

As for the potential mechanism by which ZC3H18 regulates keratins, existing studies suggest that the *ZC3H18* gene positively regulates NF-κB through the IκB kinase complex (IKK) (33), and NF-κB is known to regulate various keratins, including KRT6A and KRT14 (34). We further hypothesize that ZC3H18 may indirectly regulate the expression of cellular keratins by modulating IKK (IκB kinase). Additionally, as a transcription cofactor, ZC3H18 could regulate gene expression through other transcription factors, such as p63, SOX2, KLF4, KLF5, and KLF6, all of which may regulate cytokeratins (35). Thus, it is also possible that ZC3H18 regulates cytokeratins through these transcription factors.

In conclusion, these findings indicate that the *ZC3H18* gene has potential as a novel biomarker for esophageal cancer, with significant potential in the prognosis of the disease. Changes in ZC3H18 expression can significantly affect the tumor biological functions of esophageal cancer cells. However, there are still several limitations that should be considered. Firstly, we have not conducted experimental validation to directly examine how ZC3H18 regulates keratin expression, which may, in turn, influence tumor development. At this stage, this description remains speculative, and we plan to further explore this mechanism in future work. Secondly, we were unable to identify a single variable responsible for the regulation of *ZC3H18* gene methylation and its upstream factors. Therefore, we propose that future research could focus on the exploration of ZC3H18 inhibitors, targeted therapeutic strategies, and their clinical application value, particularly in synergy with immunotherapy. ZC3H18 is may become a specific prognostic marker and a potential therapeutic target for esophageal cancer.

## Data Availability

The original contributions presented in the study are publicly available. This data can be found here: GEO repository, accession number: GSE290209 (https://www.ncbi.nlm.nih.gov/geo/query/acc.cgi?acc=GSE290209).
