# Supplementary material for: Zinc finger protein ZC3H18 is abnormally expressed in esophageal cancer tissues and facilitates the proliferation of esophageal cancer cells
Source: Front Immunol. 2025 Feb 25;16:1556509. doi: 10.3389/fimmu.2025.1556509 (PMC11894379; doi:10.3389/fimmu.2025.1556509)
Supplement: Supplementary file 1 [file DataSheet1.zip › cell experiments/Apoptosis/data/ZYJ/231117-wangzhao2 001 00002436 025. pdf]

Institution

Protocol:

Listmode

Analysis D

Settings F

Listmode

s

Run Date: xx xxx xxxx

Sample ID: [Multiple]

User ID: user

/ 10000 (PROTOCOL)

Tube ID: NoRead

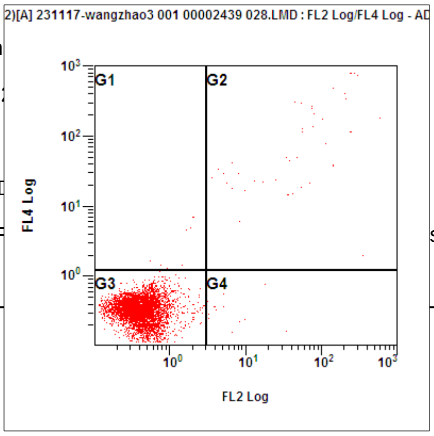

(F2)[A] 231117-wangzhao3 001 00002439 028.LMD : FL2 Log/FL4 Log					
Region	Number	%Total	%Gated	X-Mean	Y-Mean
ALL	9997	99.97	100.00	1.36	1.7
G1	17	0.17	0.17	1.55	5.27
G2	85	0.85	0.85	90.2	158
G3	9857	98.57	98.60	0.441	0.357
G4	38	0.38	0.38	41.7	0.366

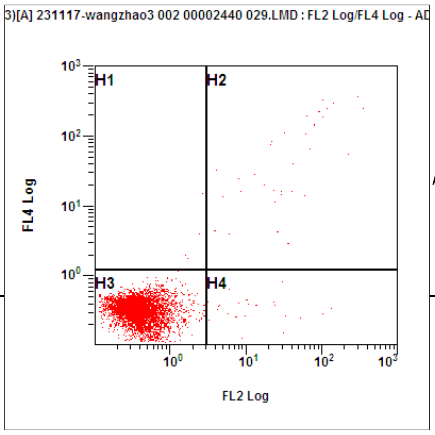

(F3)[A] 231117-wangzhao3 002 00002440 029.LMD : FL2 Log/FL4 Log					
Region	Number	%Total	%Gated	X-Mean	Y-Mean
ALL	9998	99.98	100.00	0.871	0.88
H1	14	0.14	0.14	1.91	4.67
H2	61	0.61	0.61	55.7	87
H3	9875	98.75	98.77	0.443	0.345
H4	48	0.48	0.48	19	0.393

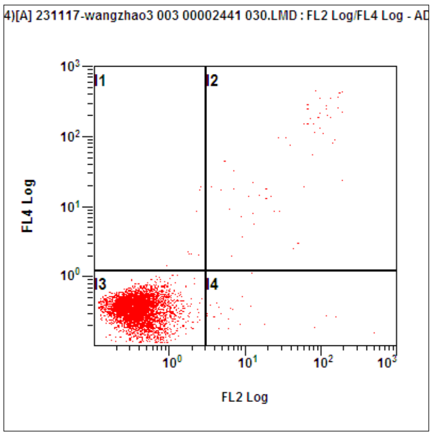

(F4)[A] 231117-wangzhao3 003 00002441 030.LMD : FL2 Log/FL4 Log					
Region	Number	%Total	%Gated	X-Mean	Y-Mean
ALL	9995	99.95	100.00	1.31	1.72
I1	27	0.27	0.27	1.48	4.51
I2	105	1.05	1.05	71.4	128
I3	9813	98.13	98.18	0.425	0.371
I4	50	0.50	0.50	28.3	0.34

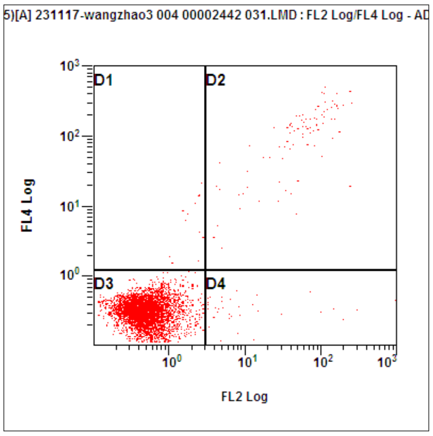

(F5)[A] 231117-wangzhao3 004 00002442 031.LMD : FL2 Log/FL4 Log					
Region	Number	%Total	%Gated	X-Mean	Y-Mean
ALL	9988	99.88	100.00	2	2.71
D1	20	0.20	0.20	1.89	7.06
D2	163	1.63	1.63	69	145
D3	9726	97.26	97.38	0.549	0.342
D4	79	0.79	0.79	42	0.376

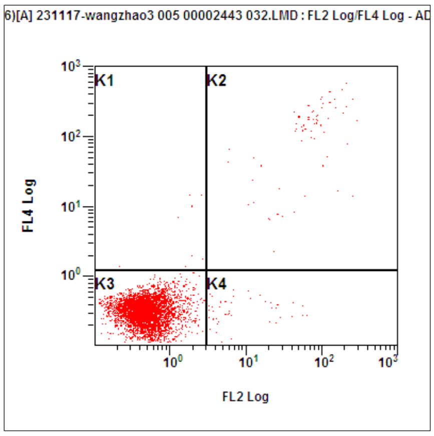

(F6)[A] 231117-wangzhao3 005 00002443 032.LMD : FL2 Log/FL4 Log					
Region	Number	%Total	%Gated	X-Mean	Y-Mean
ALL	9987	99.87	100.00	1.83	2.41
K1	20	0.20	0.20	1.91	7.53
K2	156	1.56	1.56	77.2	131
K3	9729	97.29	97.42	0.533	0.349
K4	82	0.82	0.82	12.4	0.407

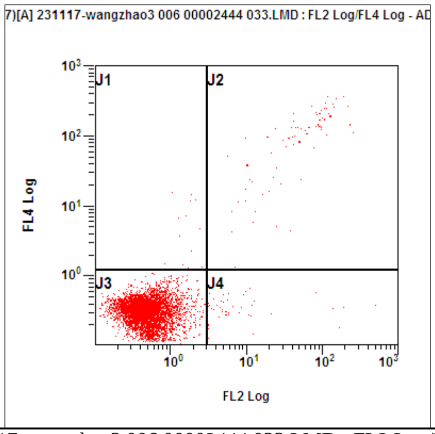

(F7)[A] 231117-wangzhao3 006 00002444 033.LMD : FL2 Log/FL4 Log					
Region	Number	%Total	%Gated	X-Mean	Y-Mean
ALL	9989	99.89	100.00	1.8	2.33
J1	28	0.28	0.28	1.61	6.58
J2	147	1.47	1.47	72.1	134
J3	9736	97.36	97.47	0.546	0.34
J4	78	0.78	0.78	25.4	0.376
