# Supplementary material for: Zinc finger protein ZC3H18 is abnormally expressed in esophageal cancer tissues and facilitates the proliferation of esophageal cancer cells
Source: Front Immunol. 2025 Feb 25;16:1556509. doi: 10.3389/fimmu.2025.1556509 (PMC11894379; doi:10.3389/fimmu.2025.1556509)
Supplement: Supplementary file 1 [file DataSheet1.zip › cell experiments/Apoptosis/data/ZYJ/231117-wangzhao2 002 00002436 025. pdf. pdf]

Institution

Protocol:

Listmode

Analysis D

Settings F

Listmode

Run Date: xx xxx xxxx

Sample ID: [Multiple]

User ID: user

/ 10000 (PROTOCOL)

Tube ID: NoRead

s

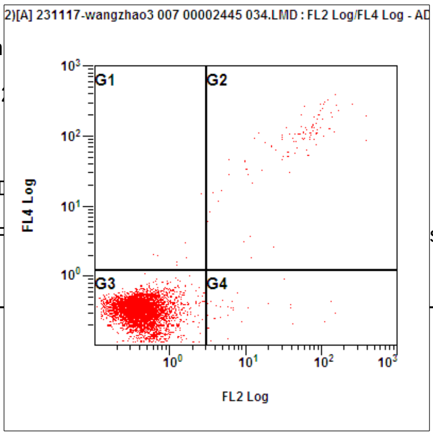

(F2)[A] 231117-wangzhao3 007 00002445 034.LMD : FL2 Log/FL4 Log					
Region	Number	%Total	%Gated	X-Mean	Y-Mean
ALL	9974	99.74	100.00	1.95	2.76
G1	12	0.12	0.12	1.64	4.42
G2	187	1.87	1.87	70.7	129
G3	9725	97.25	97.50	0.461	0.353
G4	50	0.50	0.50	33.8	0.365

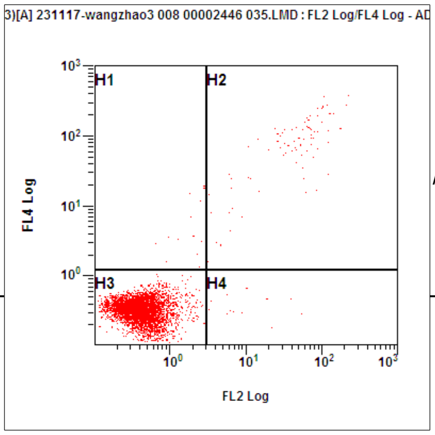

(F3)[A] 231117-wangzhao3 008 00002446 035.LMD : FL2 Log/FL4 Log					
Region	Number	%Total	%Gated	X-Mean	Y-Mean
ALL	9982	99.82	100.00	1.87	2.41
H1	18	0.18	0.18	1.76	6.44
H2	195	1.95	1.95	64.7	105
H3	9729	97.29	97.47	0.451	0.35
H4	40	0.40	0.40	39.9	0.376

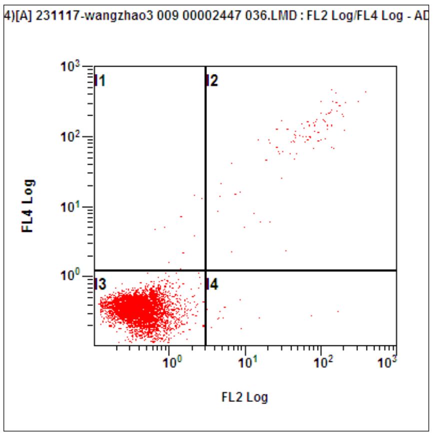

(F4)[A] 231117-wangzhao3 009 00002447 036.LMD : FL2 Log/FL4 Log					
Region	Number	%Total	%Gated	X-Mean	Y-Mean
ALL	9979	99.79	100.00	1.84	2.59
I1	24	0.24	0.24	1.66	5.44
I2	172	1.72	1.72	77.2	128
I3	9751	97.51	97.72	0.443	0.369
I4	32	0.32	0.32	22.9	0.297

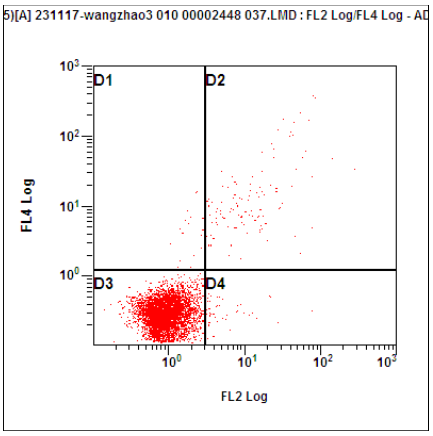

(F5)[A] 231117-wangzhao3 010 00002448 037.LMD : FL2 Log/FL4 Log					
Region	Number	%Total	%Gated	X-Mean	Y-Mean
ALL	9954	99.54	100.00	1.57	1.03
D1	49	0.49	0.49	2.09	5.89
D2	227	2.27	2.28	20.4	30.2
D3	9566	95.66	96.10	0.985	0.321
D4	112	1.12	1.13	13	0.399

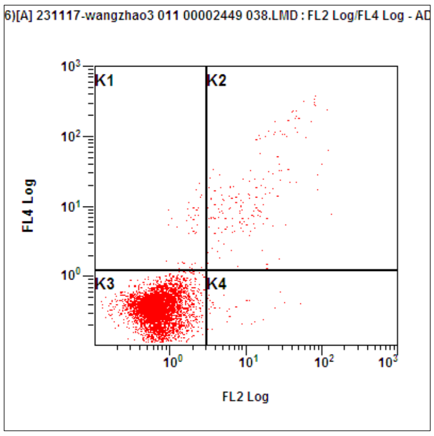

(F6)[A] 231117-wangzhao3 011 00002449 038.LMD : FL2 Log/FL4 Log					
Region	Number	%Total	%Gated	X-Mean	Y-Mean
ALL	9958	99.58	100.00	1.43	1.81
K1	88	0.88	0.88	1.85	5.61
K2	307	3.07	3.08	19.9	45.3
K3	9489	94.89	95.29	0.767	0.384
K4	74	0.74	0.74	9.76	0.363

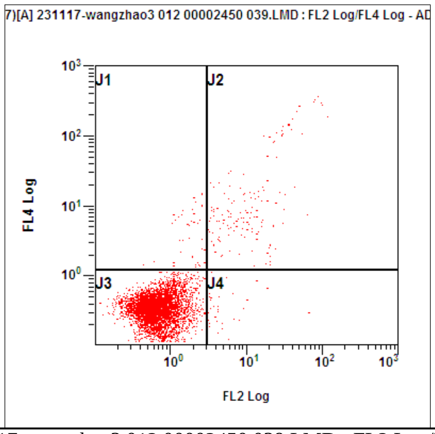

(F7)[A] 231117-wangzhao3 012 00002450 039.LMD : FL2 Log/FL4 Log					
Region	Number	%Total	%Gated	X-Mean	Y-Mean
ALL	9958	99.58	100.00	1.32	1.58
J1	80	0.80	0.80	1.88	4.33
J2	303	3.03	3.04	17.5	38.9
J3	9475	94.75	95.15	0.74	0.378
J4	100	1.00	1.00	6.94	0.404
