# Supplementary material for: Zinc finger protein ZC3H18 is abnormally expressed in esophageal cancer tissues and facilitates the proliferation of esophageal cancer cells
Source: Front Immunol. 2025 Feb 25;16:1556509. doi: 10.3389/fimmu.2025.1556509 (PMC11894379; doi:10.3389/fimmu.2025.1556509)
Supplement: Supplementary file 1 [file DataSheet1.zip › cell experiments/Apoptosis/data/ZYJ/231117-wangzhao2 003 00002436 025. pdf. pdf]

Institution

Protocol: :

Listmode

Analysis D

Settings F s

Listmode

Run Date: xx xxx xxxx

Sample ID: [Multiple]

User ID: user

/ 10000 (PROTOCOL)

Tube ID: NoRead

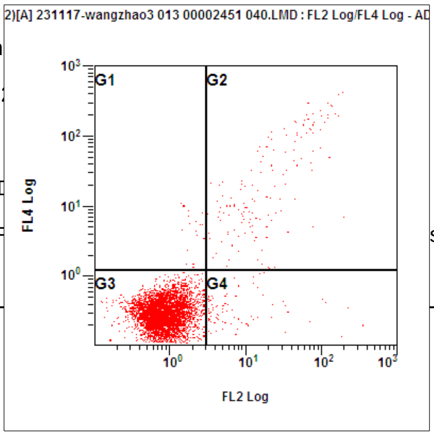

(F2)[A] 231117-wangzhao3 013 00002451 040.LMD : FL2 Log/FL4 Log

Region	Number	%Total	%Gated	X-Mean	Y-Mean
ALL	9924	99.24	100.00	2.22	2.24
G1	52	0.52	0.52	1.89	4.64
G2	265	2.65	2.67	39.5	71.3
G3	9430	94.30	95.02	0.915	0.323
G4	177	1.77	1.78	16.2	0.419

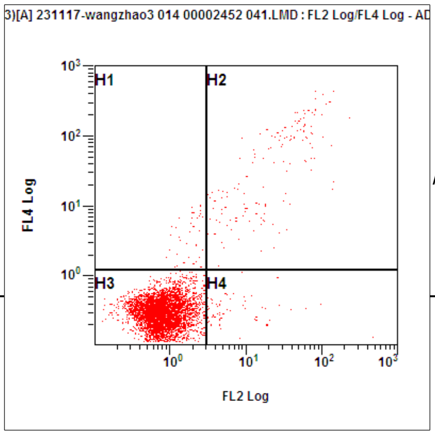

(F3)[A] 231117-wangzhao3 014 00002452 041.LMD : FL2 Log/FL4 Log

Region	Number	%Total	%Gated	X-Mean	Y-Mean
ALL	9892	98.92	100.00	2.09	2.57
H1	81	0.81	0.82	1.97	5.11
H2	273	2.73	2.76	38.7	80.1
H3	9402	94.02	95.05	0.882	0.331
H4	136	1.36	1.37	12.5	0.388

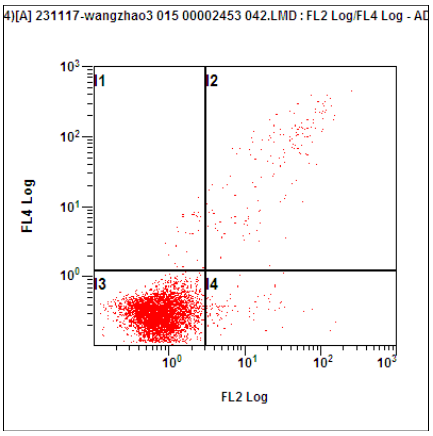

(F4)[A] 231117-wangzhao3 015 00002453 042.LMD : FL2 Log/FL4 Log

Region	Number	%Total	%Gated	X-Mean	Y-Mean
ALL	9910	99.10	100.00	2.33	3.06
I1	59	0.59	0.60	1.83	4.33
I2	297	2.97	3.00	42.9	90.6
I3	9365	93.65	94.50	0.856	0.323
I4	189	1.89	1.91	11.8	0.409

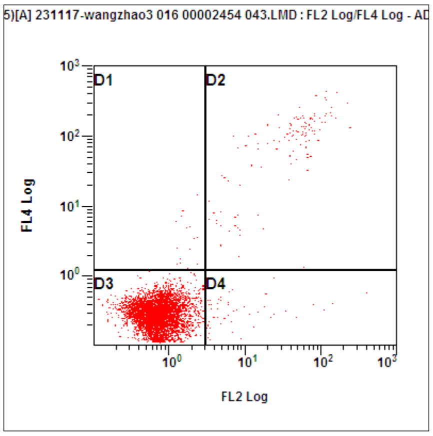

(F5)[A] 231117-wangzhao3 016 00002454 043.LMD : FL2 Log/FL4 Log

Region	Number	%Total	%Gated	X-Mean	Y-Mean
ALL	9944	99.44	100.00	2.27	3.16
D1	40	0.40	0.40	1.86	4.38
D2	235	2.35	2.36	52.2	120
D3	9564	95.64	96.18	0.772	0.313
D4	105	1.05	1.06	27.4	0.382

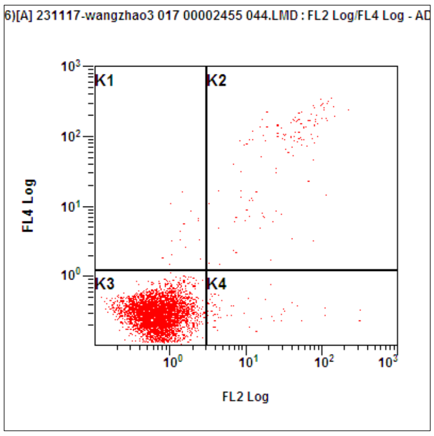

(F6)[A] 231117-wangzhao3 017 00002455 044.LMD : FL2 Log/FL4 Log

Region	Number	%Total	%Gated	X-Mean	Y-Mean
ALL	9964	99.64	100.00	2.1	2.89
K1	28	0.28	0.28	1.71	5.27
K2	227	2.27	2.28	49.4	112
K3	9610	96.10	96.45	0.761	0.322
K4	99	0.99	0.99	24	0.336

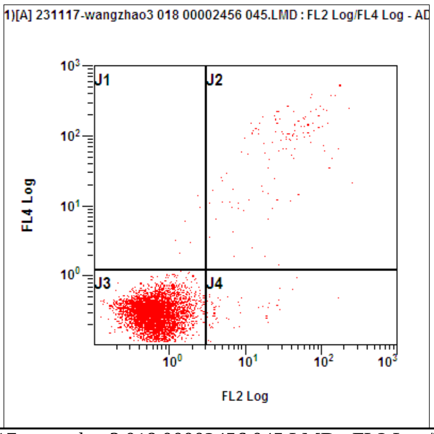

(F1)[A] 231117-wangzhao3 018 00002456 045.LMD : FL2 Log/FL4 Log

Region	Number	%Total	%Gated	X-Mean	Y-Mean
ALL	9958	99.58	100.00	1.83	2.91
J1	26	0.26	0.26	1.88	7.46
J2	227	2.27	2.28	42.5	113
J3	9588	95.88	96.28	0.719	0.329
J4	117	1.17	1.17	13.8	0.373

In
