# Supplementary figures and images for: Zinc finger protein ZC3H18 is abnormally expressed in esophageal cancer tissues and facilitates the proliferation of esophageal cancer cells
Source: Front Immunol. 2025 Feb 25;16:1556509. doi: 10.3389/fimmu.2025.1556509 (PMC11894379; doi:10.3389/fimmu.2025.1556509)

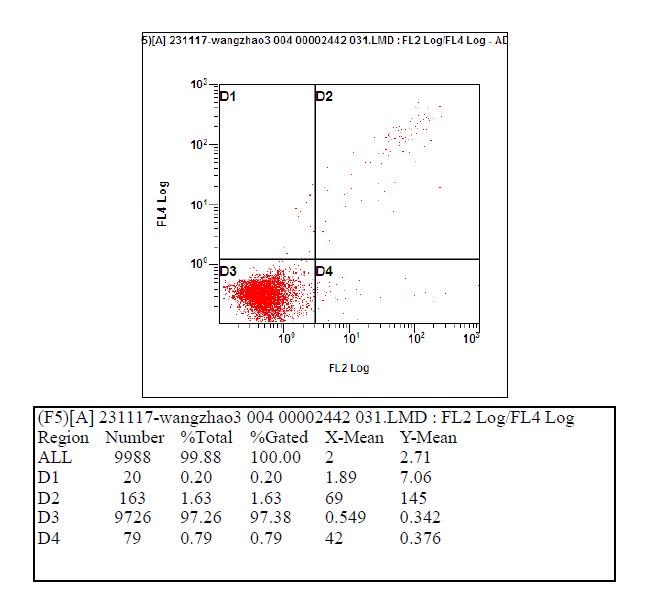

Supplement: Supplementary file 1 [file DataSheet1.zip › cell experiments/Apoptosis/ECA109/si-1/1.jpg]

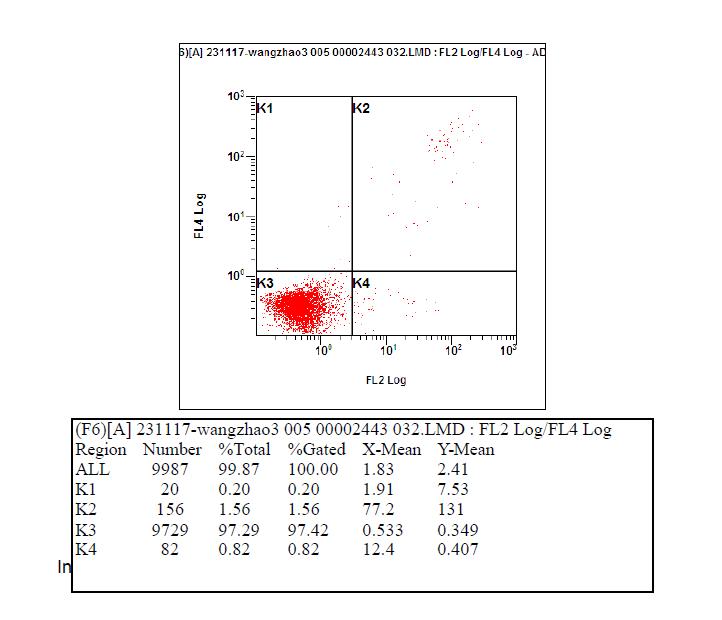

Supplement: Supplementary file 1 [file DataSheet1.zip › cell experiments/Apoptosis/ECA109/si-1/2.jpg]

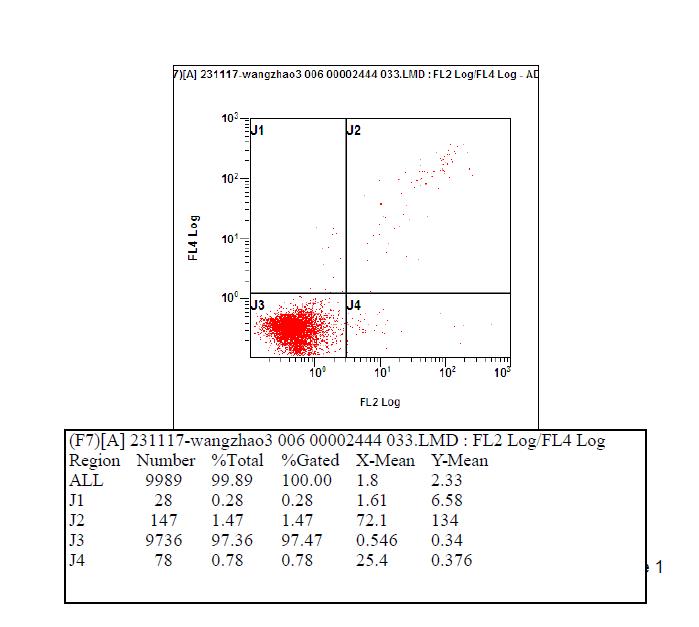

Supplement: Supplementary file 1 [file DataSheet1.zip › cell experiments/Apoptosis/ECA109/si-1/3.jpg]

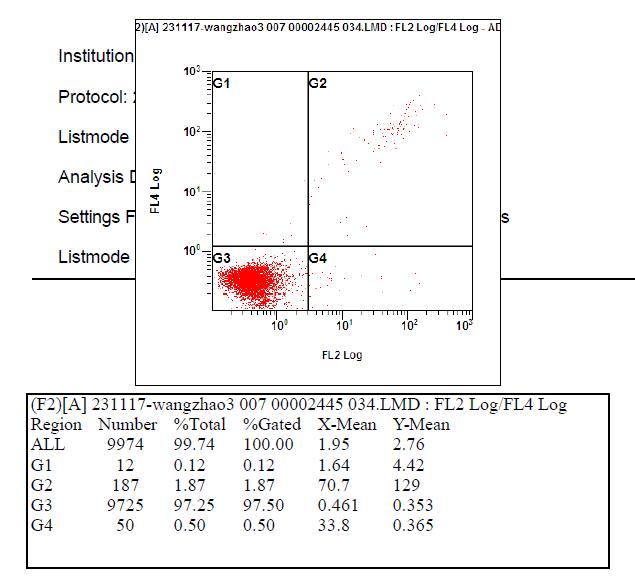

Supplement: Supplementary file 1 [file DataSheet1.zip › cell experiments/Apoptosis/ECA109/si-2/1.jpg]

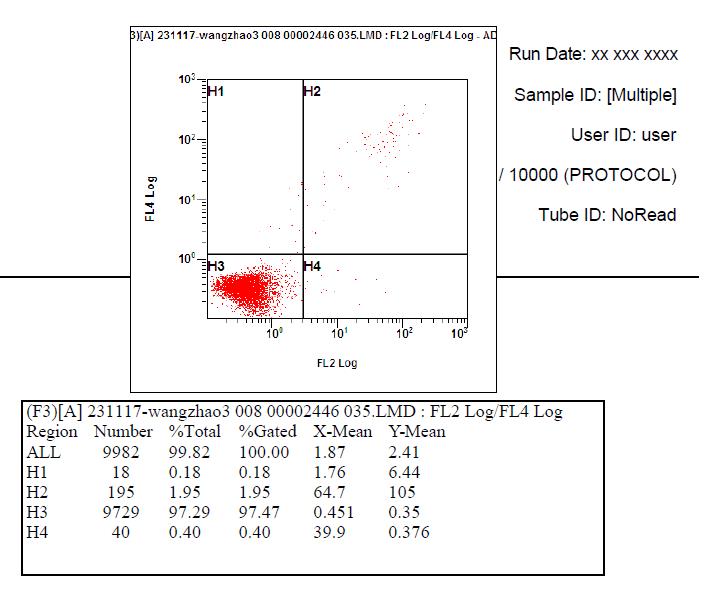

Supplement: Supplementary file 1 [file DataSheet1.zip › cell experiments/Apoptosis/ECA109/si-2/2.jpg]

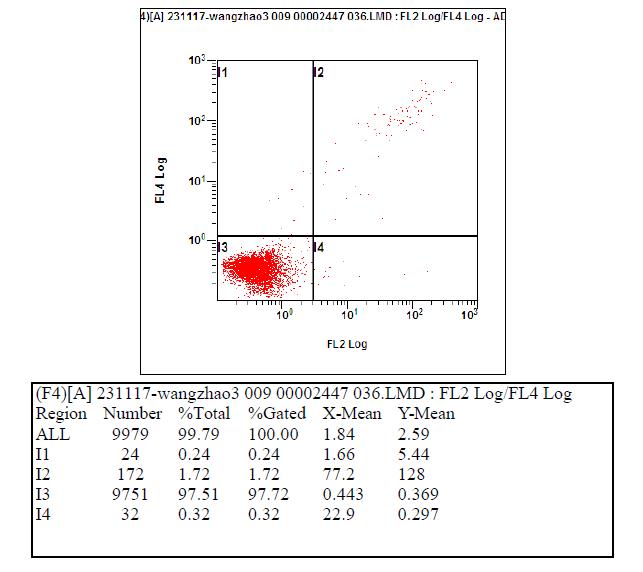

Supplement: Supplementary file 1 [file DataSheet1.zip › cell experiments/Apoptosis/ECA109/si-2/3.jpg]

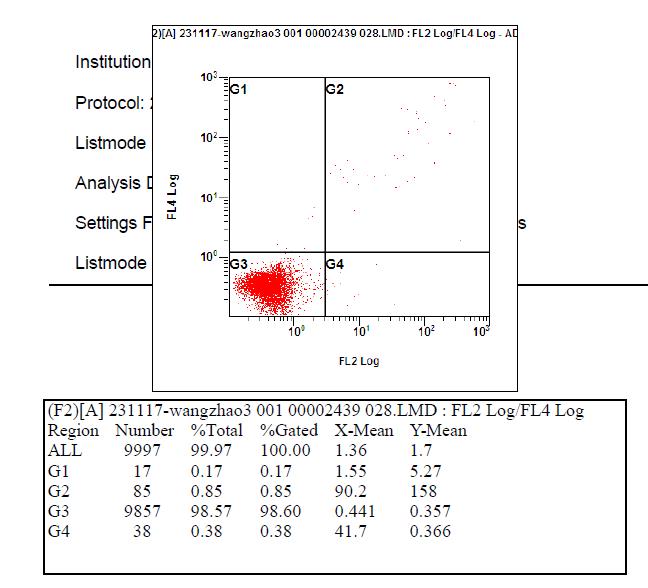

Supplement: Supplementary file 1 [file DataSheet1.zip › cell experiments/Apoptosis/ECA109/si-nc/1.jpg]

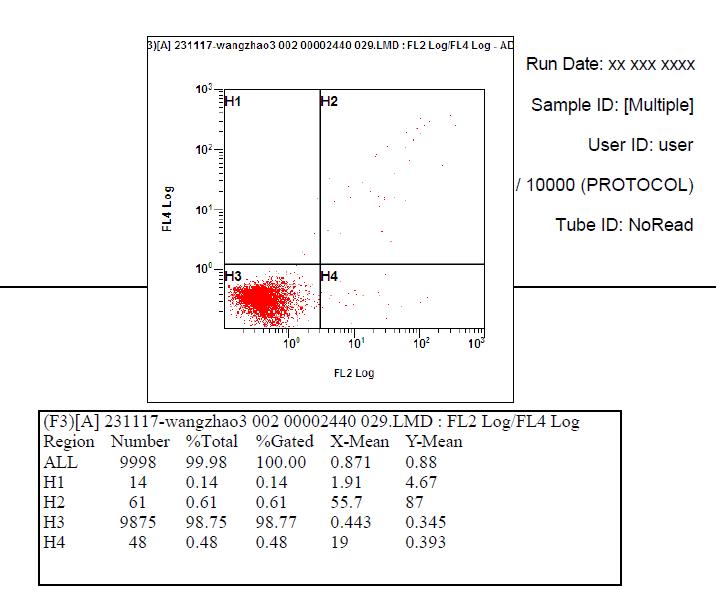

Supplement: Supplementary file 1 [file DataSheet1.zip › cell experiments/Apoptosis/ECA109/si-nc/2.jpg]

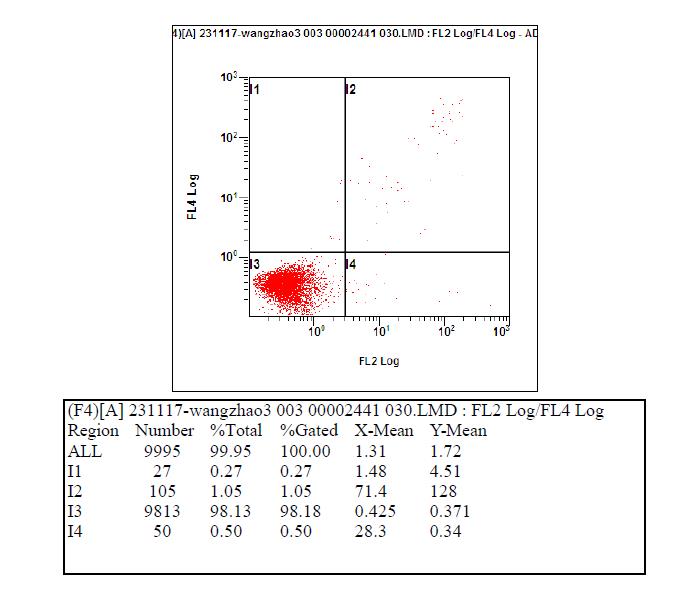

Supplement: Supplementary file 1 [file DataSheet1.zip › cell experiments/Apoptosis/ECA109/si-nc/3.jpg]

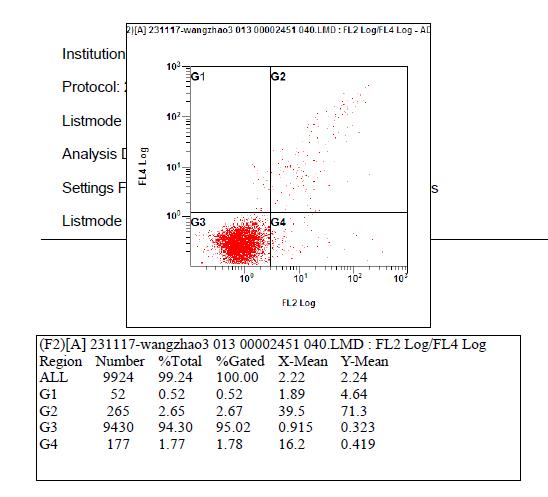

Supplement: Supplementary file 1 [file DataSheet1.zip › cell experiments/Apoptosis/KYSE150/si-1/1.jpg]

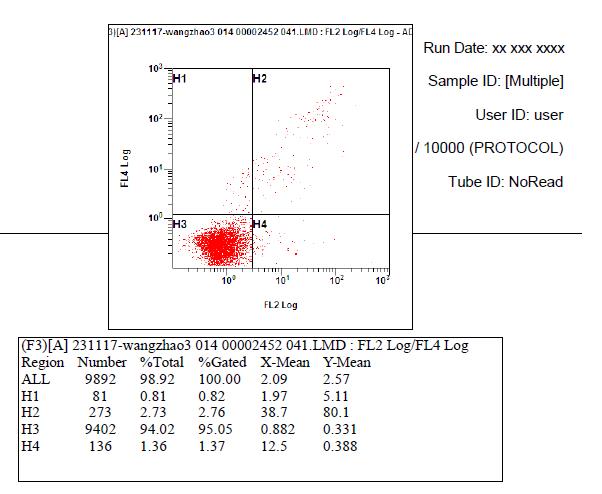

Supplement: Supplementary file 1 [file DataSheet1.zip › cell experiments/Apoptosis/KYSE150/si-1/2.jpg]

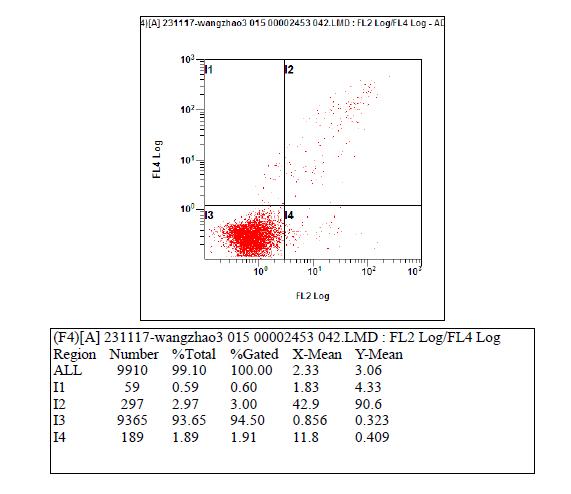

Supplement: Supplementary file 1 [file DataSheet1.zip › cell experiments/Apoptosis/KYSE150/si-1/3.jpg]

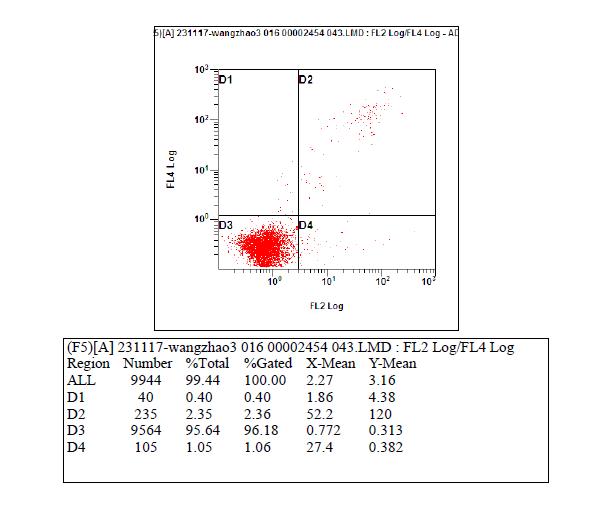

Supplement: Supplementary file 1 [file DataSheet1.zip › cell experiments/Apoptosis/KYSE150/si-2/1.jpg]

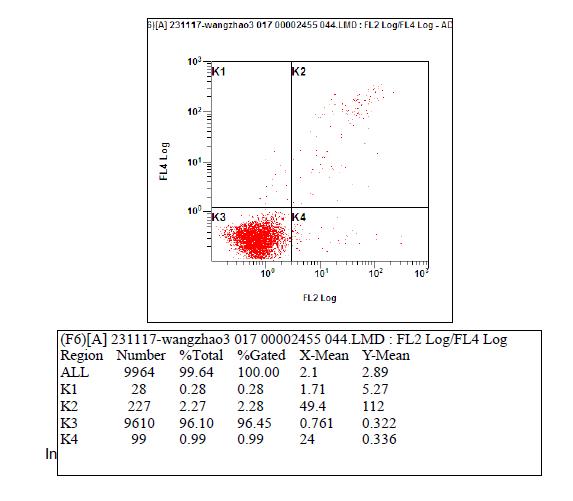

Supplement: Supplementary file 1 [file DataSheet1.zip › cell experiments/Apoptosis/KYSE150/si-2/2.jpg]

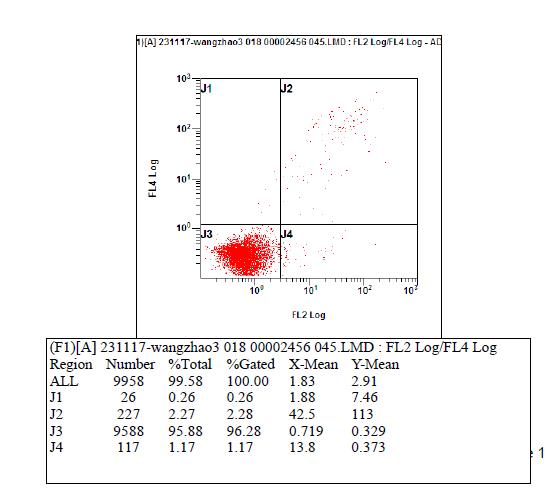

Supplement: Supplementary file 1 [file DataSheet1.zip › cell experiments/Apoptosis/KYSE150/si-2/3.jpg]

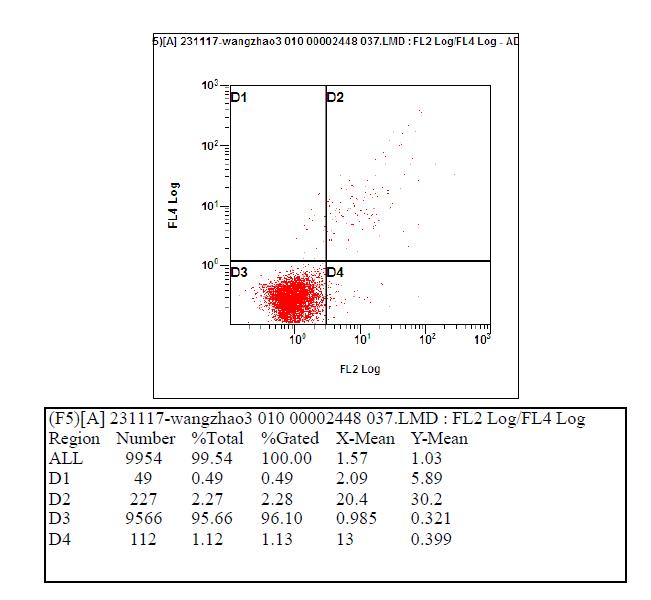

Supplement: Supplementary file 1 [file DataSheet1.zip › cell experiments/Apoptosis/KYSE150/si-nc/1.jpg]

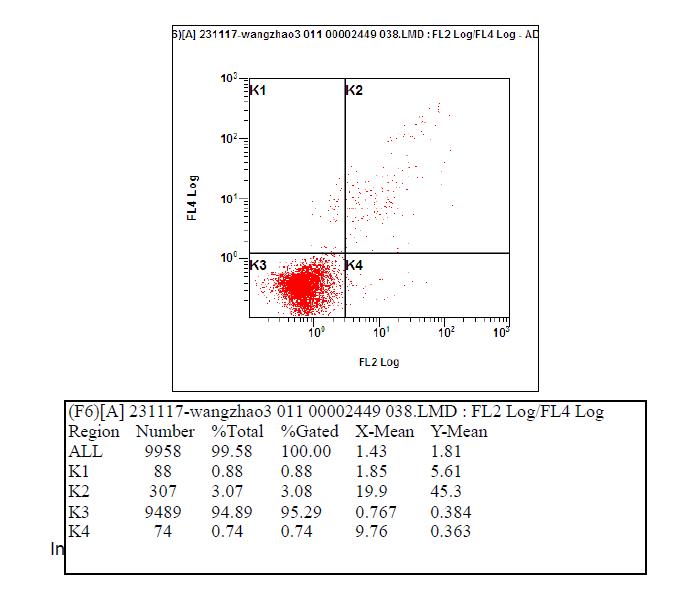

Supplement: Supplementary file 1 [file DataSheet1.zip › cell experiments/Apoptosis/KYSE150/si-nc/2.jpg]

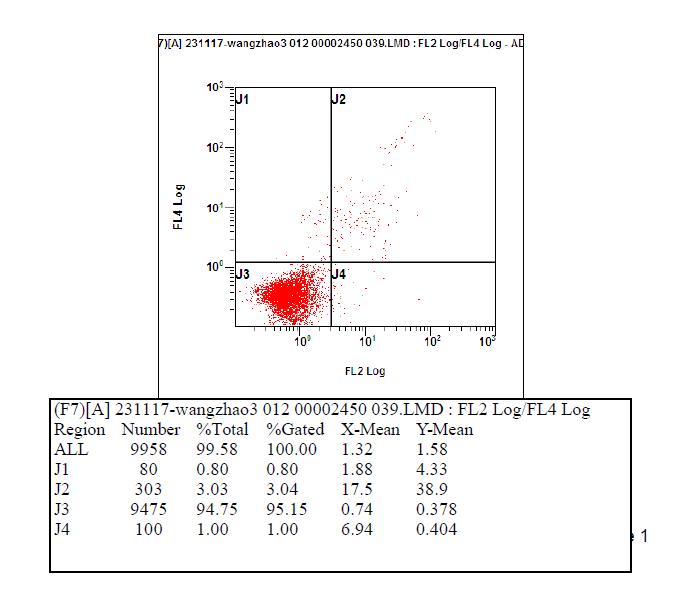

Supplement: Supplementary file 1 [file DataSheet1.zip › cell experiments/Apoptosis/KYSE150/si-nc/3.jpg]

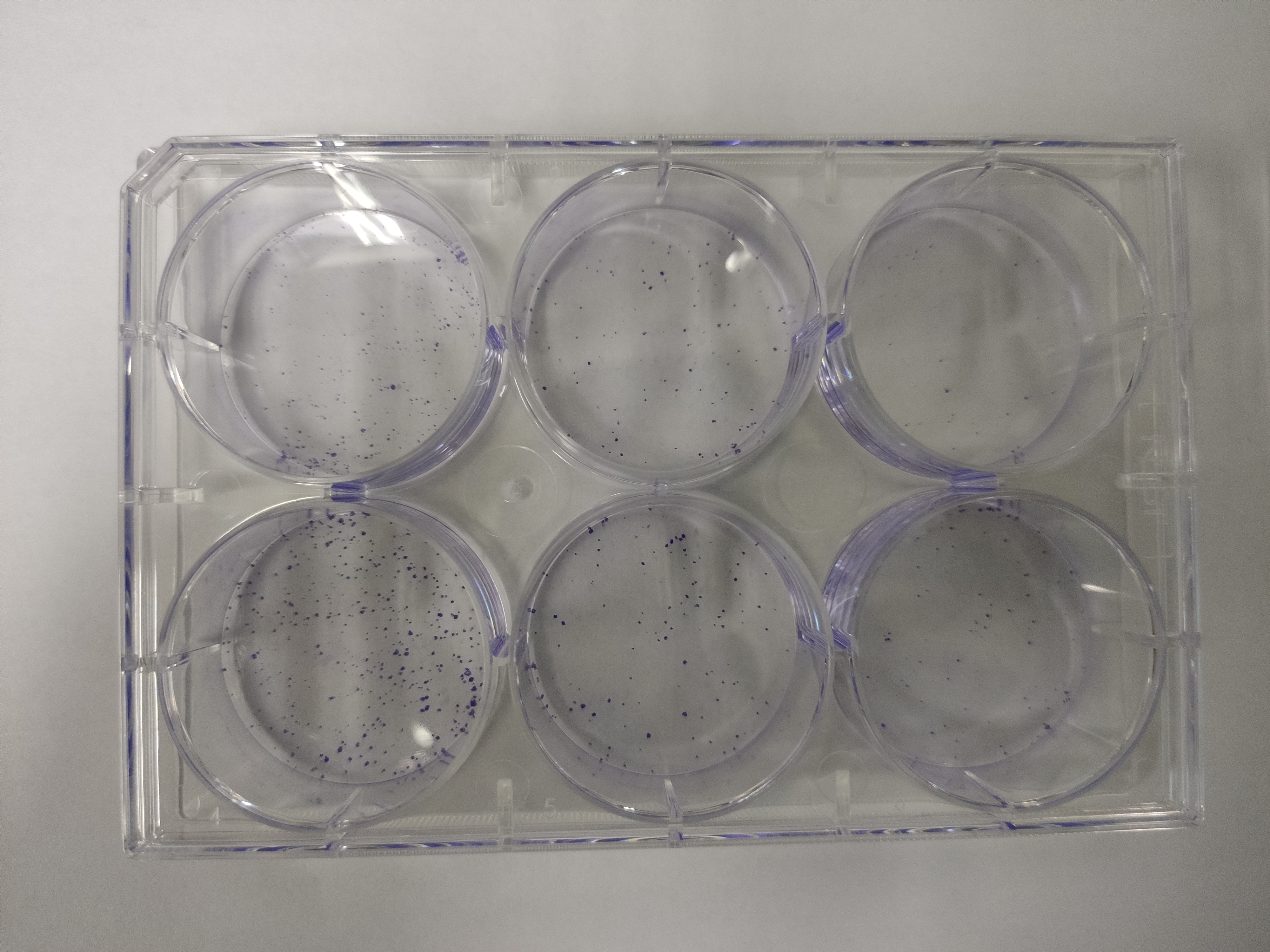

Supplement: Supplementary file 1 [file DataSheet1.zip › cell experiments/Colony formation/0.jpg]

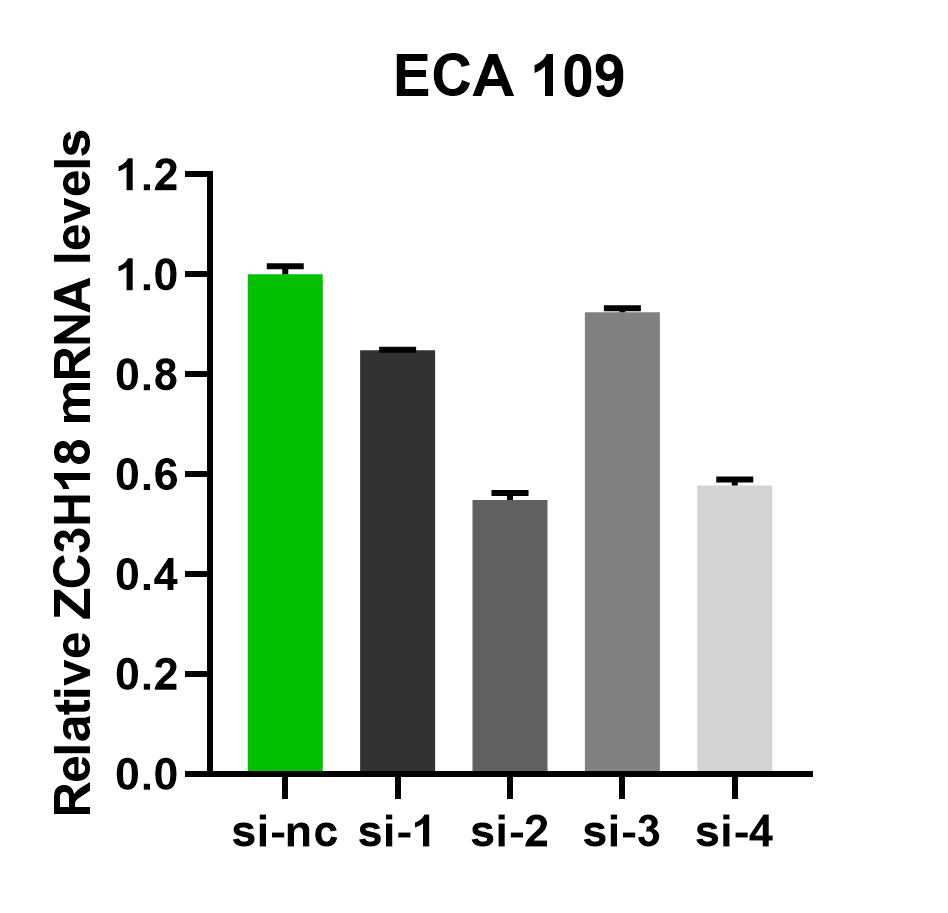

Supplement: Supplementary file 1 [file DataSheet1.zip › cell experiments/qPCR/2023-10-25/ECA109.tif]

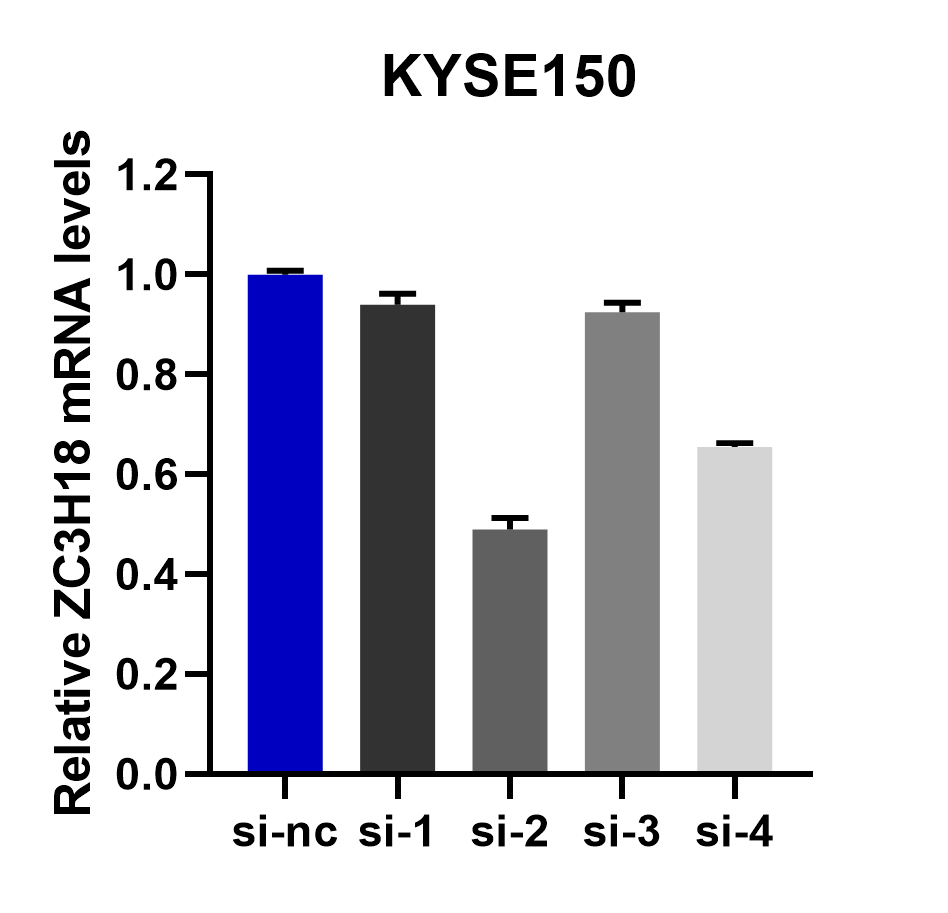

Supplement: Supplementary file 1 [file DataSheet1.zip › cell experiments/qPCR/2023-10-25/KYSE150.tif]

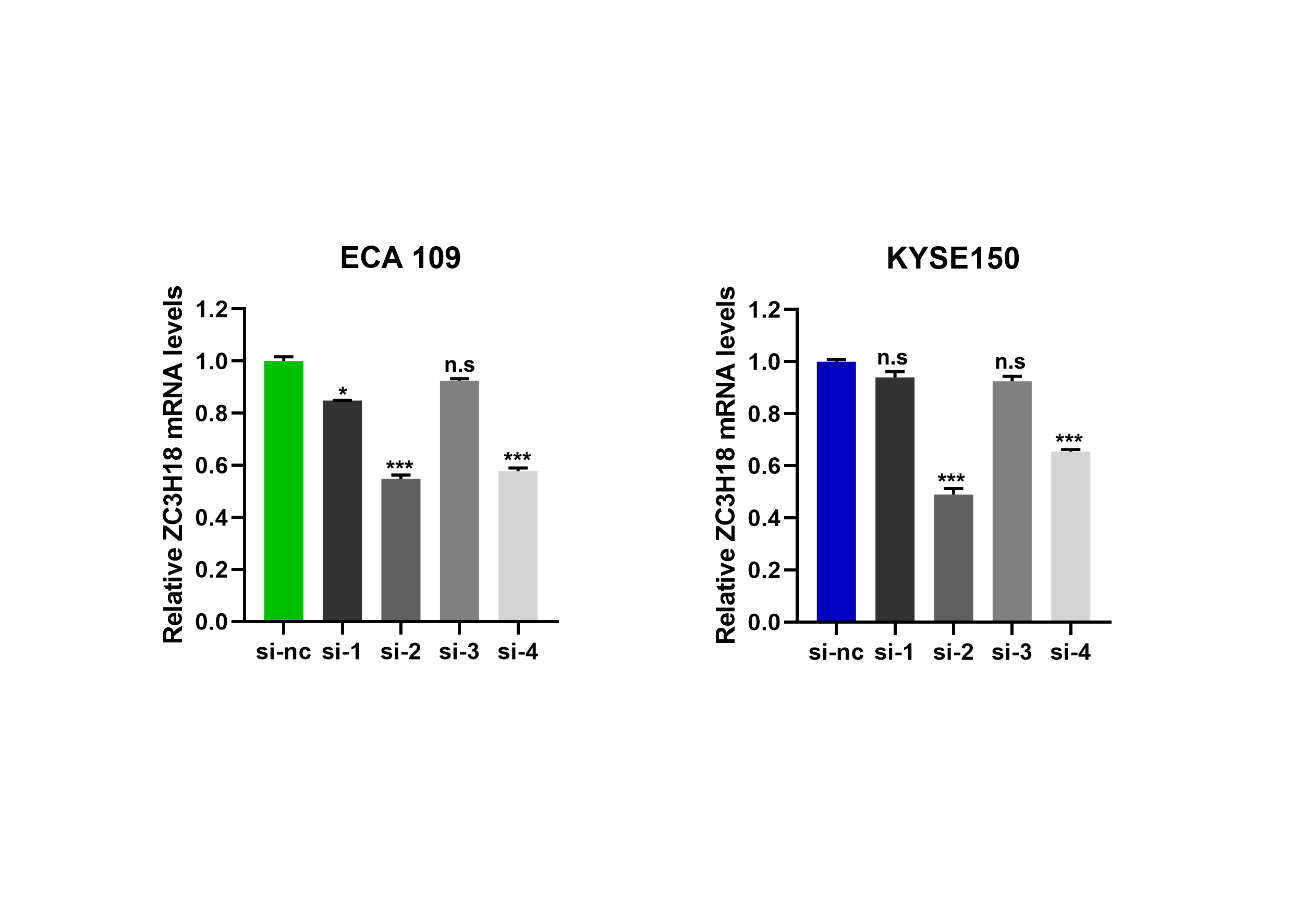

Supplement: Supplementary file 1 [file DataSheet1.zip › cell experiments/qPCR/2023-10-25/未标题-1.tif]

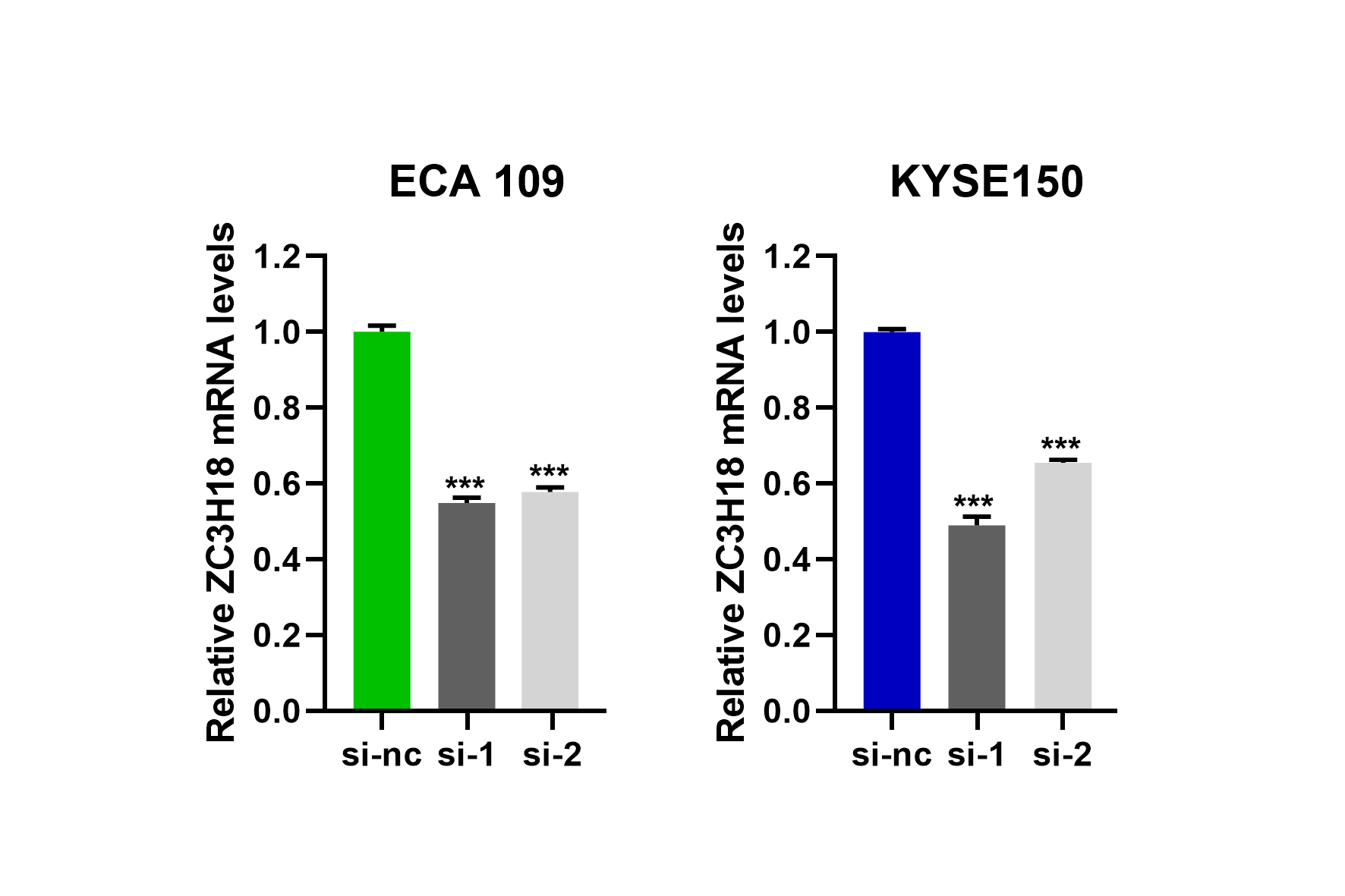

Supplement: Supplementary file 1 [file DataSheet1.zip › cell experiments/qPCR/2023-10-25/未标题-2.tif]

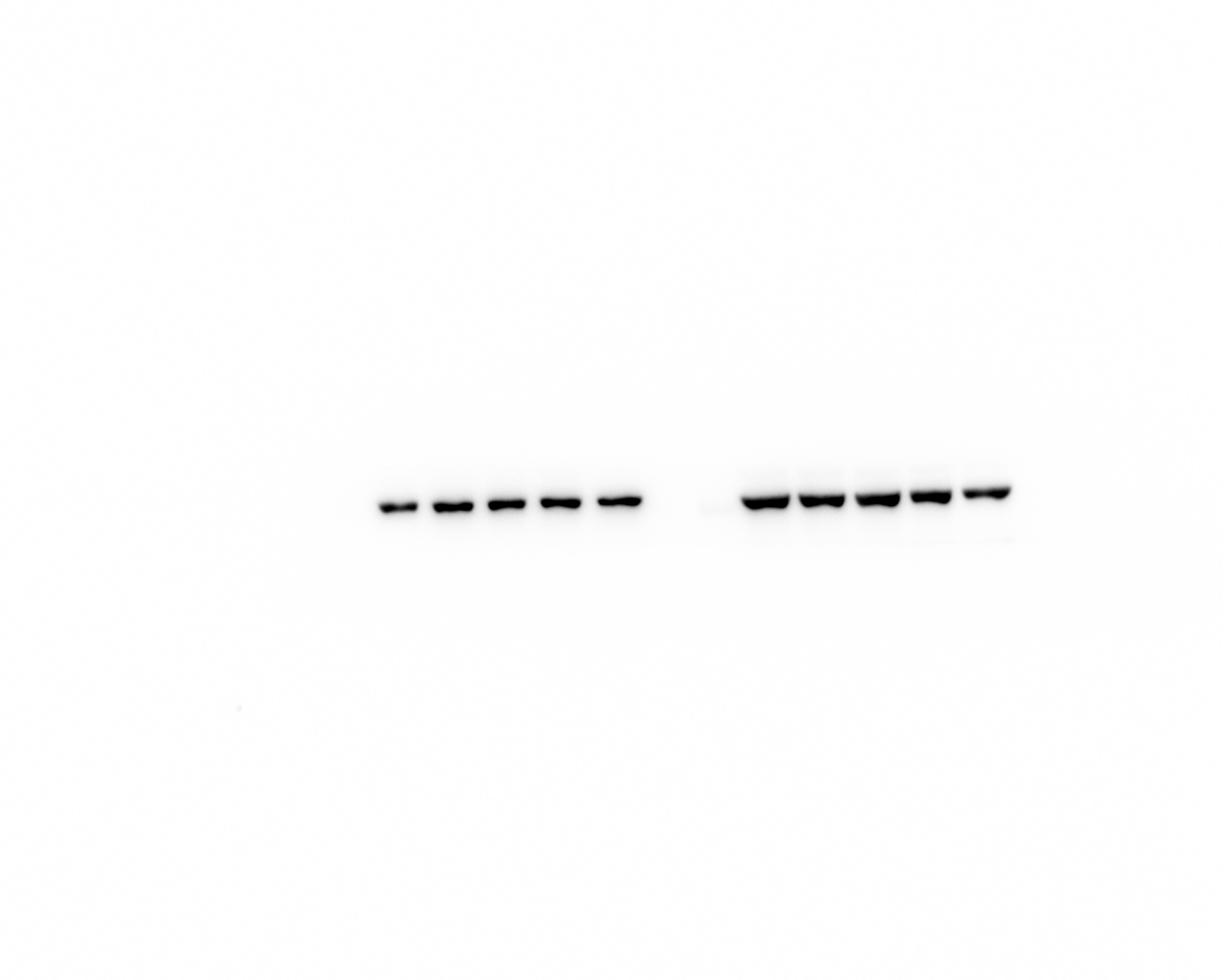

Supplement: Supplementary file 1 [file DataSheet1.zip › cell experiments/Western blot/2023-10-24/B-ACTIN.Tif]

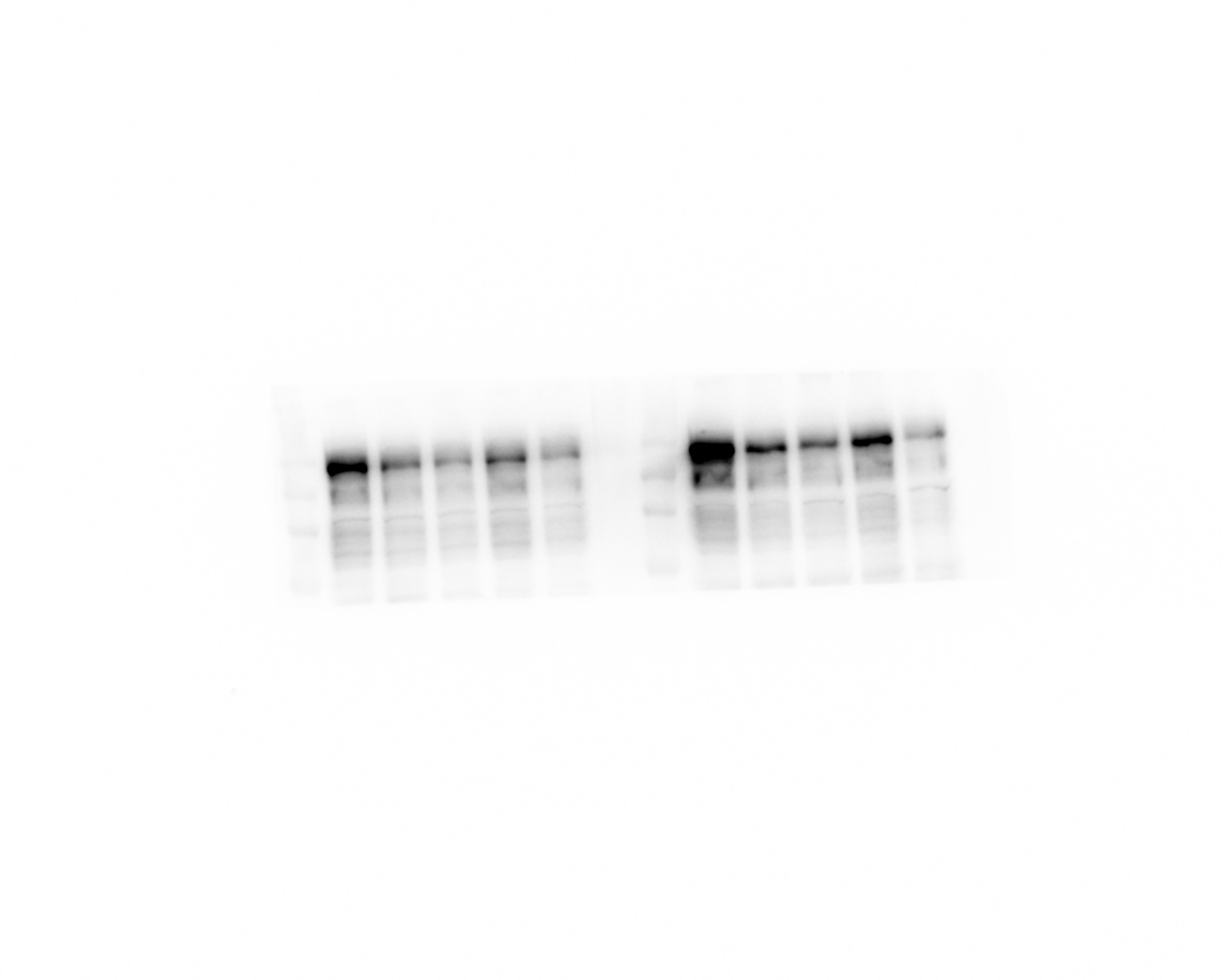

Supplement: Supplementary file 1 [file DataSheet1.zip › cell experiments/Western blot/2023-10-24/ZC3H18.jpg]

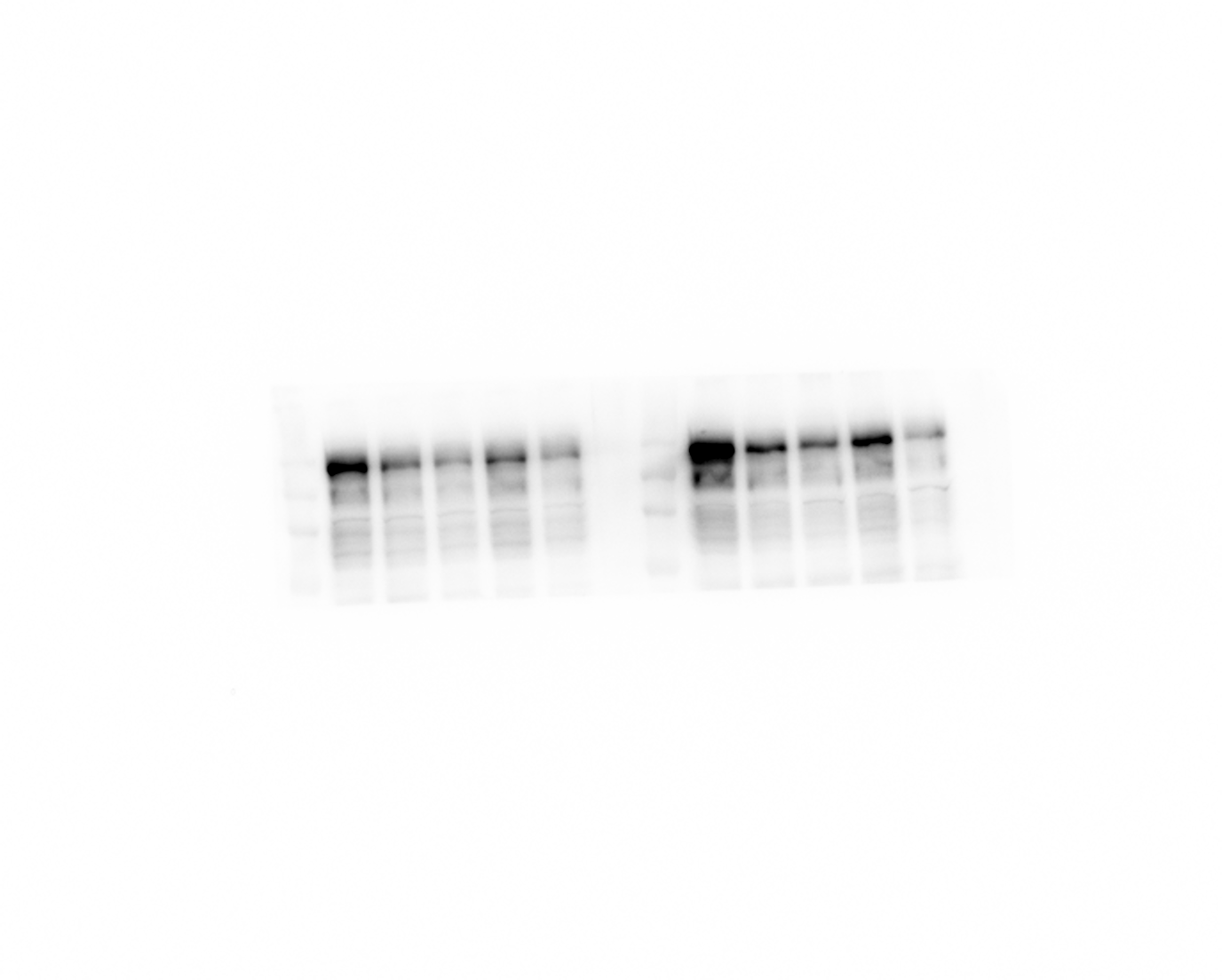

Supplement: Supplementary file 1 [file DataSheet1.zip › cell experiments/Western blot/2023-10-24/ZC3H18.Tif]

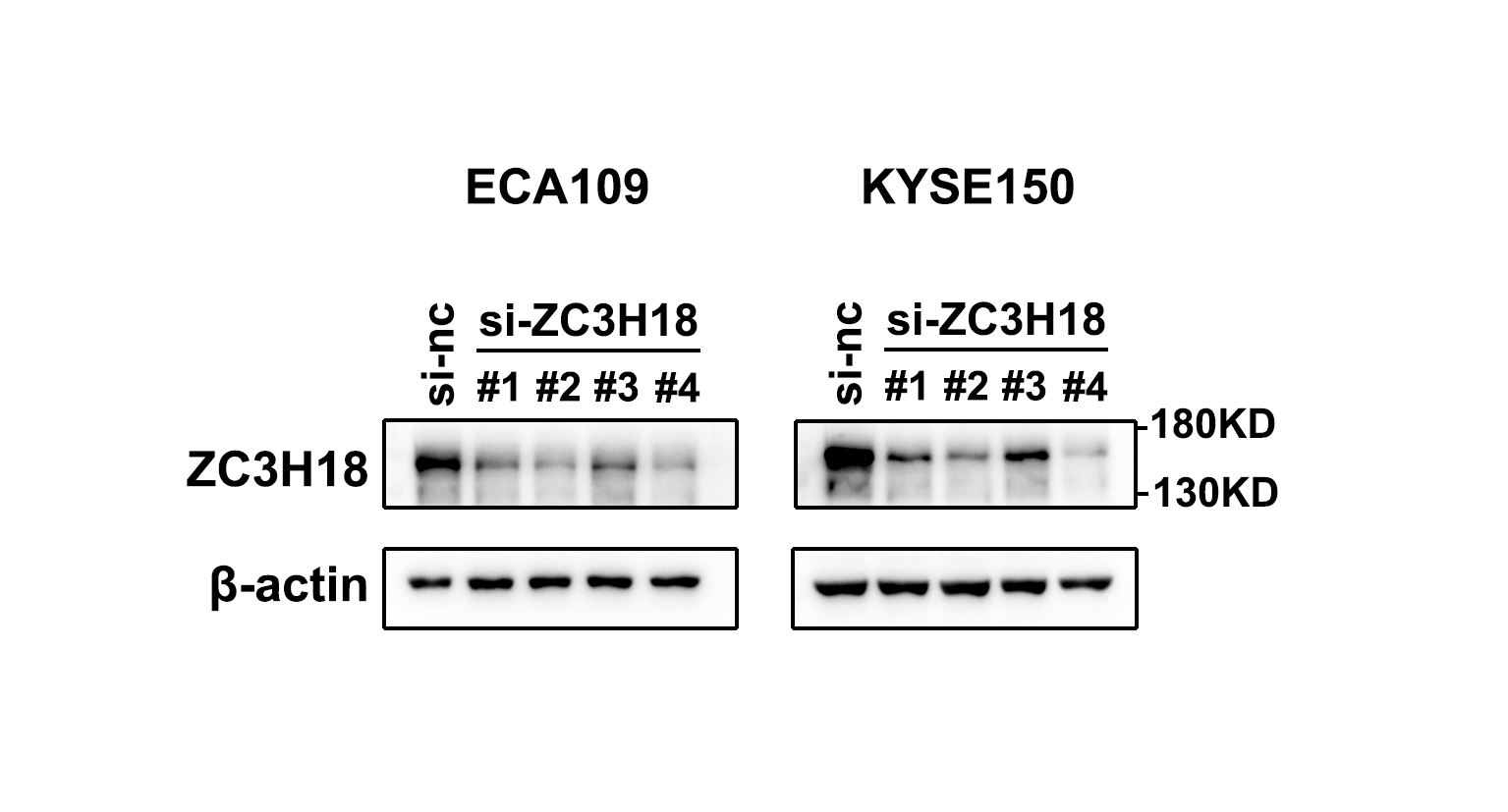

Supplement: Supplementary file 1 [file DataSheet1.zip › cell experiments/Western blot/2023-10-24/未标题-1.tif]

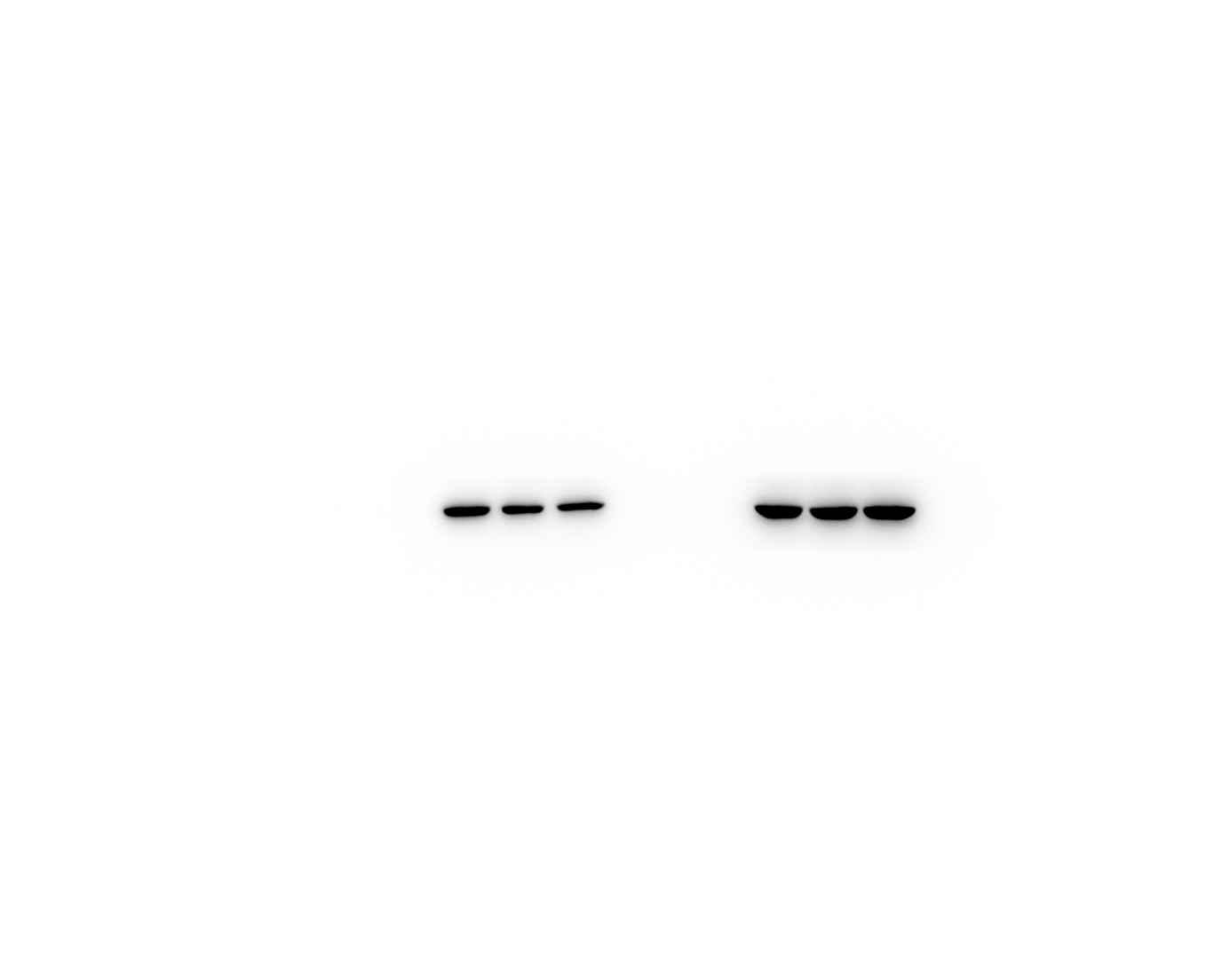

Supplement: Supplementary file 1 [file DataSheet1.zip › cell experiments/Western blot/2023-10-26/B-ACTIN-1.Tif]

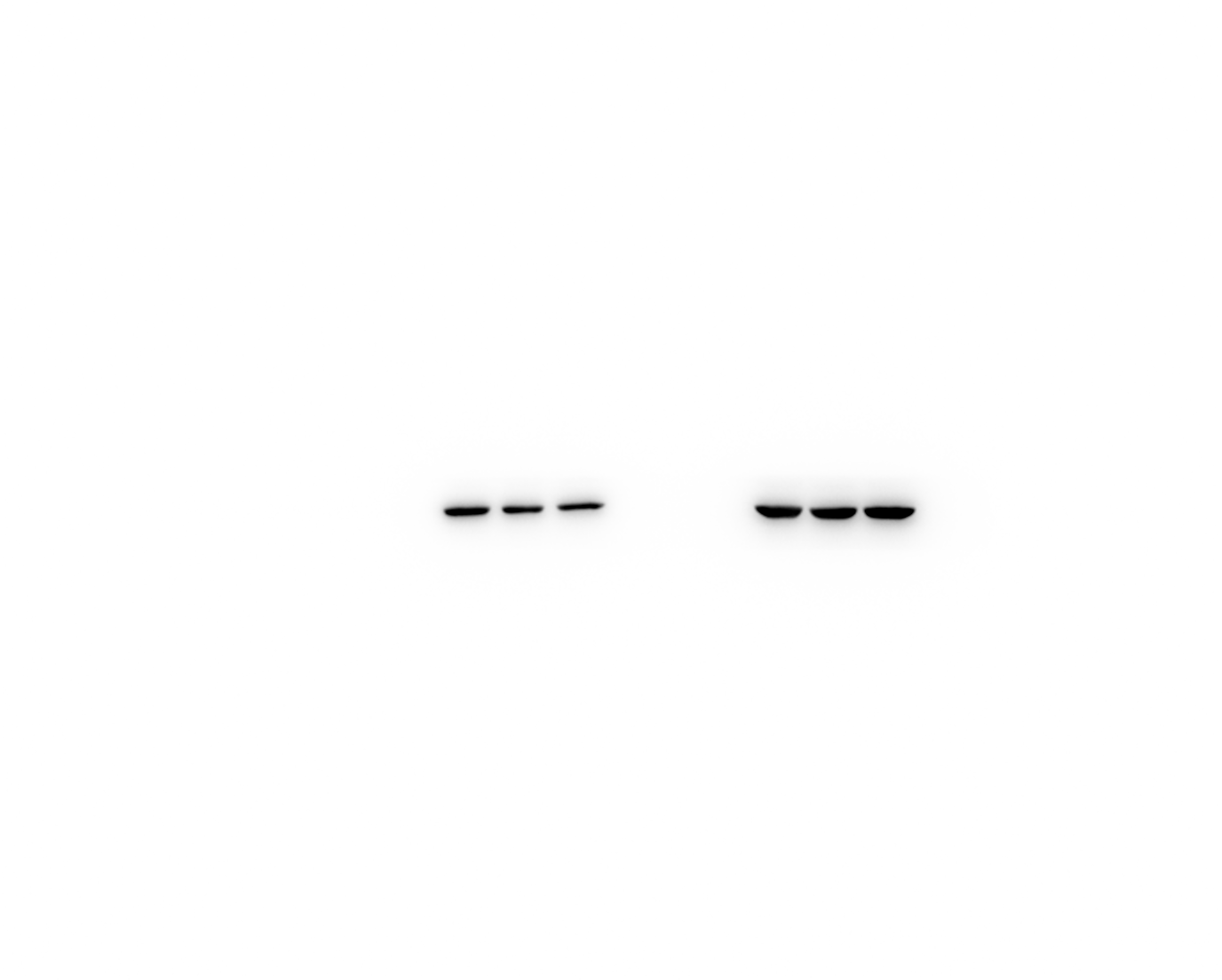

Supplement: Supplementary file 1 [file DataSheet1.zip › cell experiments/Western blot/2023-10-26/B-ACTIN-2.Tif]

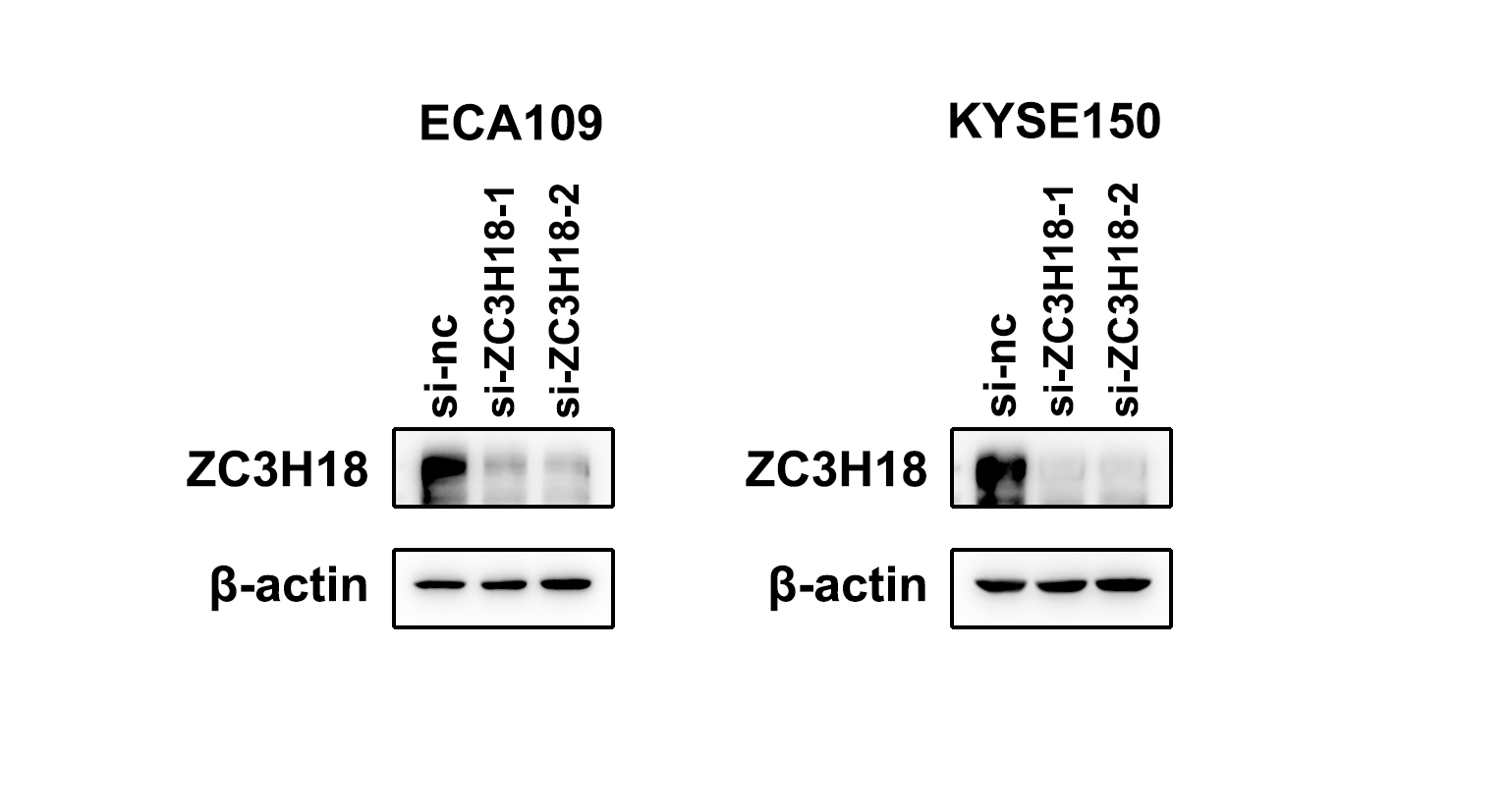

Supplement: Supplementary file 1 [file DataSheet1.zip › cell experiments/Western blot/2023-10-26/siZC3H18.tif]

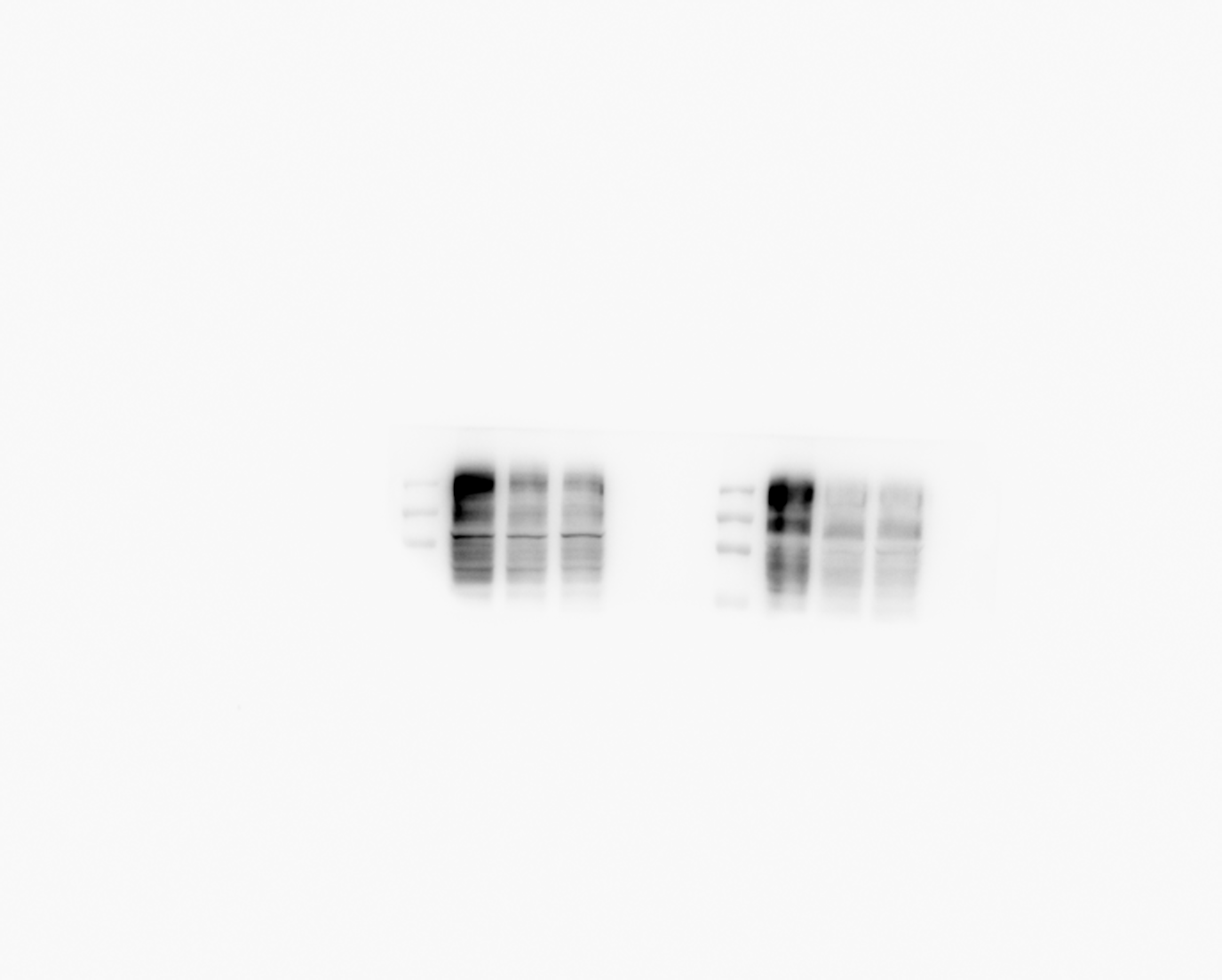

Supplement: Supplementary file 1 [file DataSheet1.zip › cell experiments/Western blot/2023-10-26/Z3CH18-1.Tif]

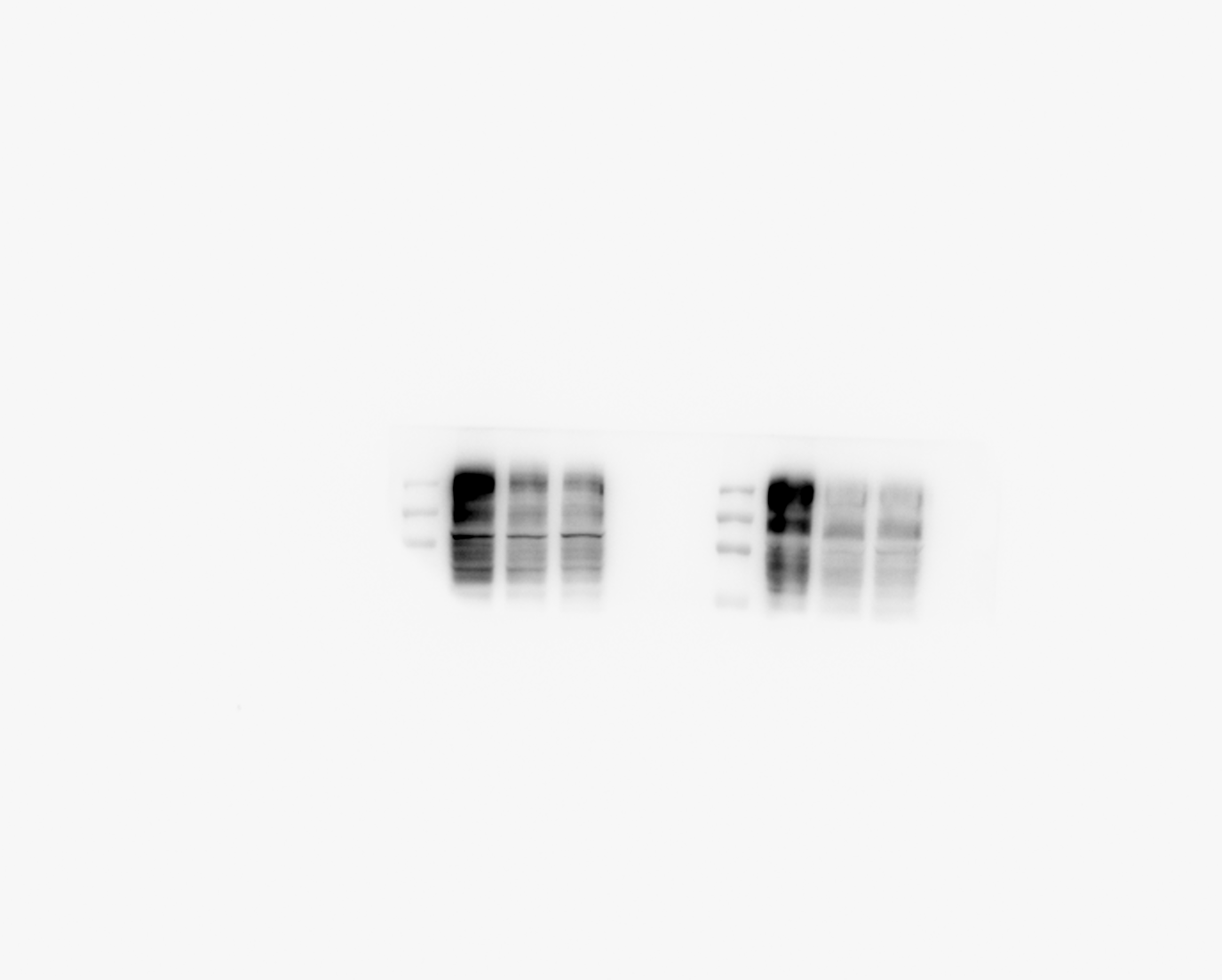

Supplement: Supplementary file 1 [file DataSheet1.zip › cell experiments/Western blot/2023-10-26/Z3CH18-2.Tif]

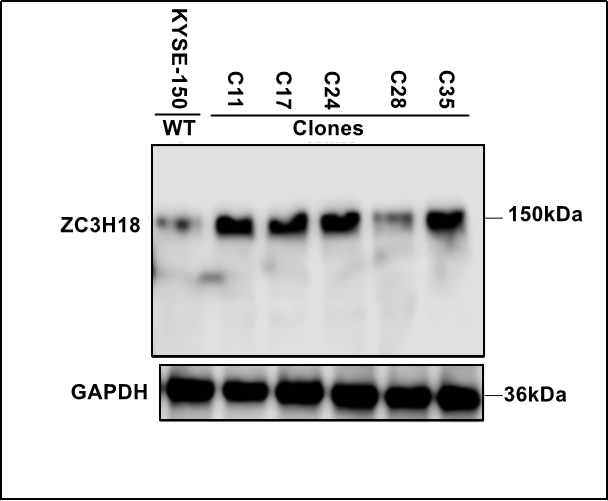

Supplement: Supplementary file 1 [file DataSheet1.zip › cell experiments/Western blot/ZC3H18 over expression.png]

PC score distribution

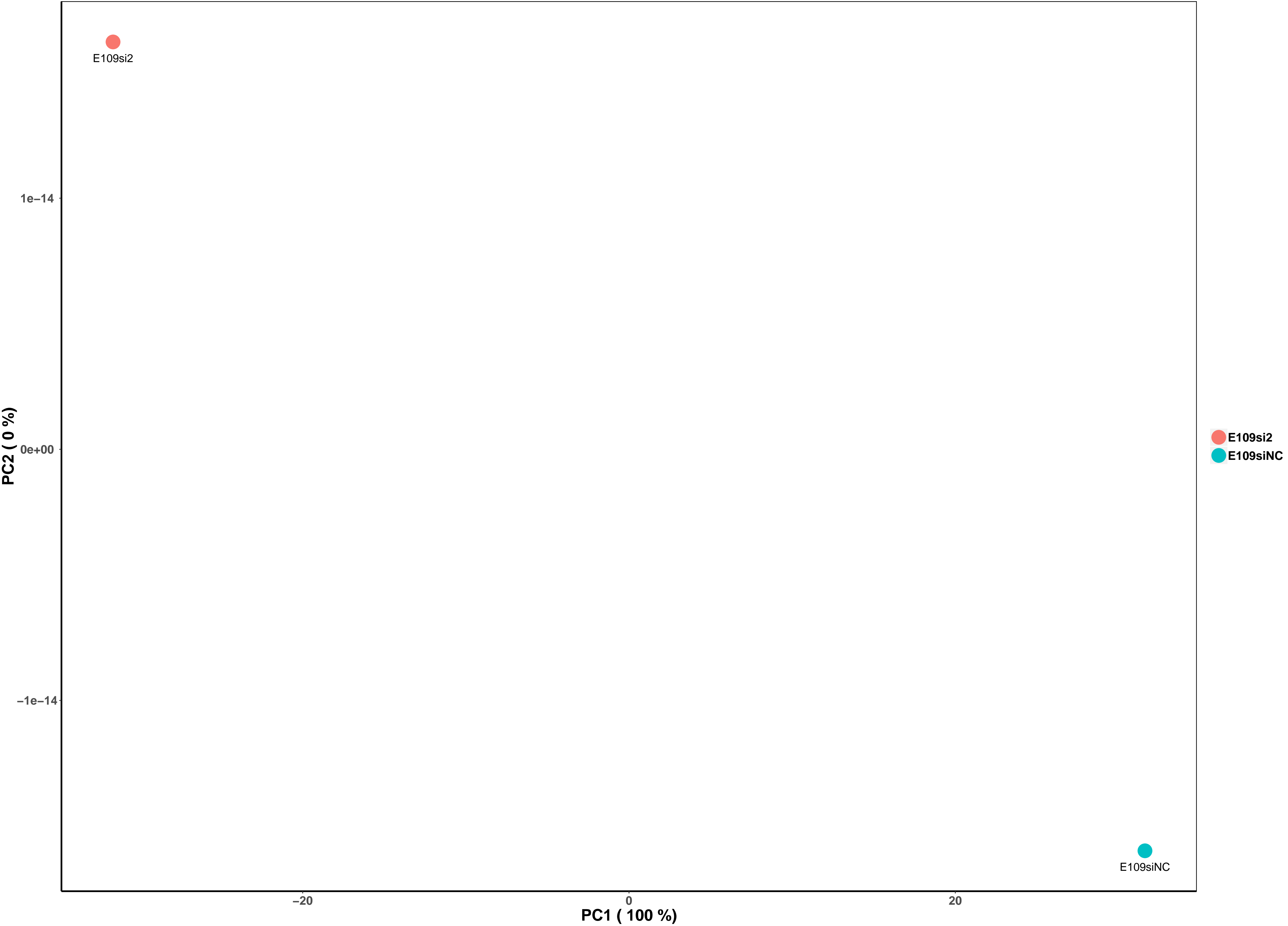

Supplement: Supplementary file 1 [file DataSheet1.zip › Results of eukaryotic transcriptome sequencing/genes/E109si2_vs_E109siNC/genes_PCA_score.pdf]

# E109si2\_vs\_E109siNC

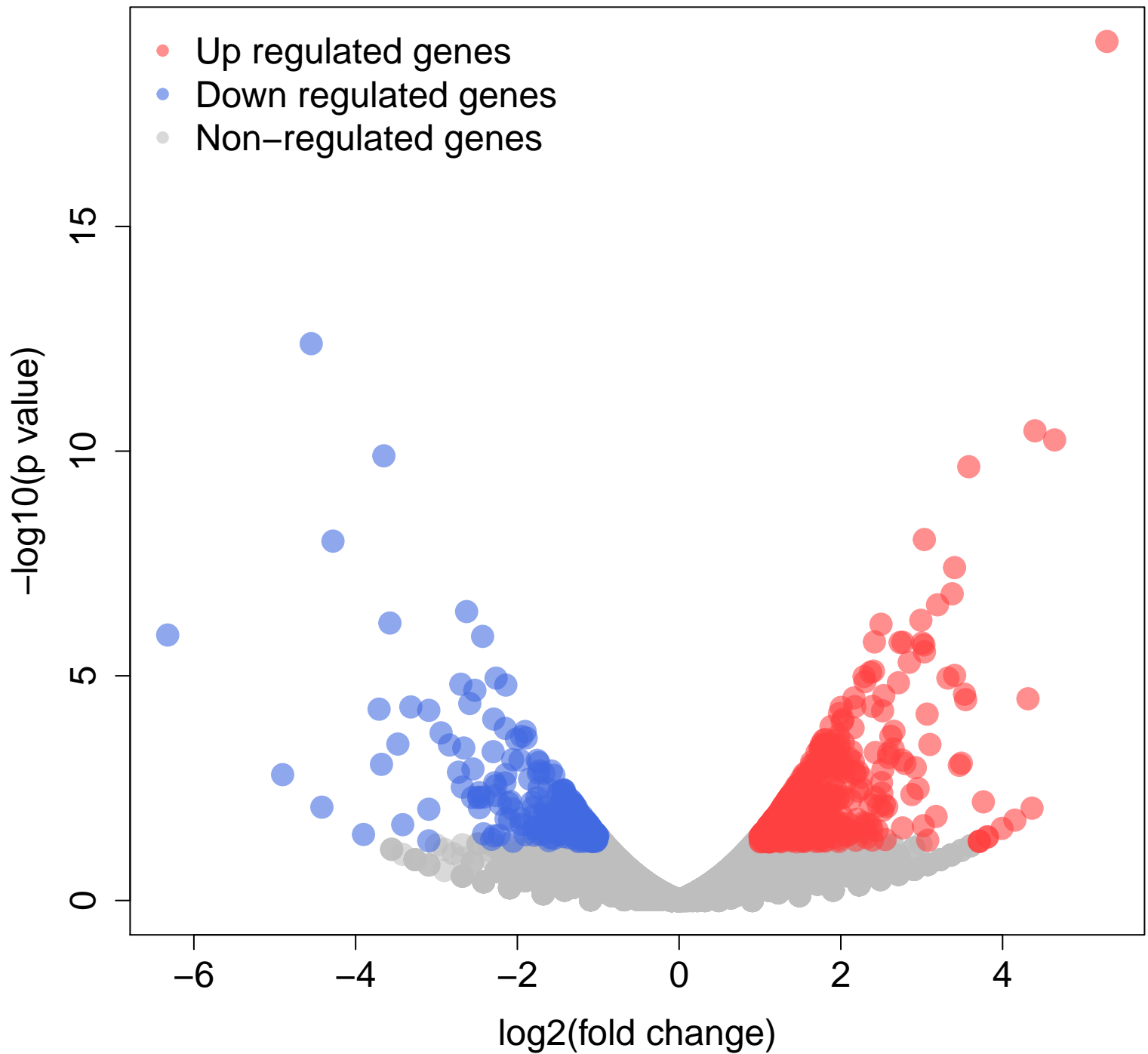

Supplement: Supplementary file 1 [file DataSheet1.zip › Results of eukaryotic transcriptome sequencing/genes/E109si2_vs_E109siNC/valcano.pdf]

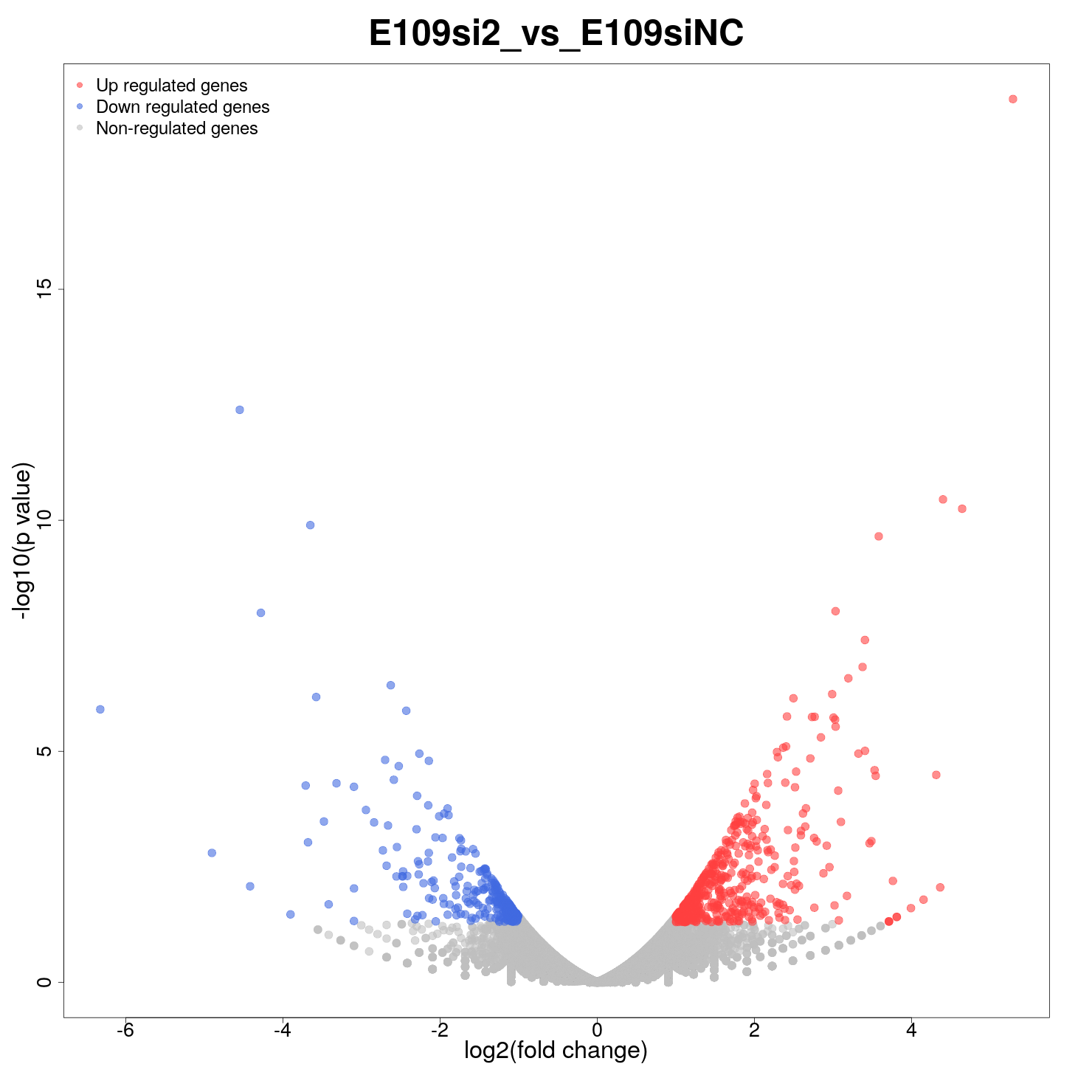

Supplement: Supplementary file 1 [file DataSheet1.zip › Results of eukaryotic transcriptome sequencing/genes/E109si2_vs_E109siNC/valcano.png]

PC score distribution

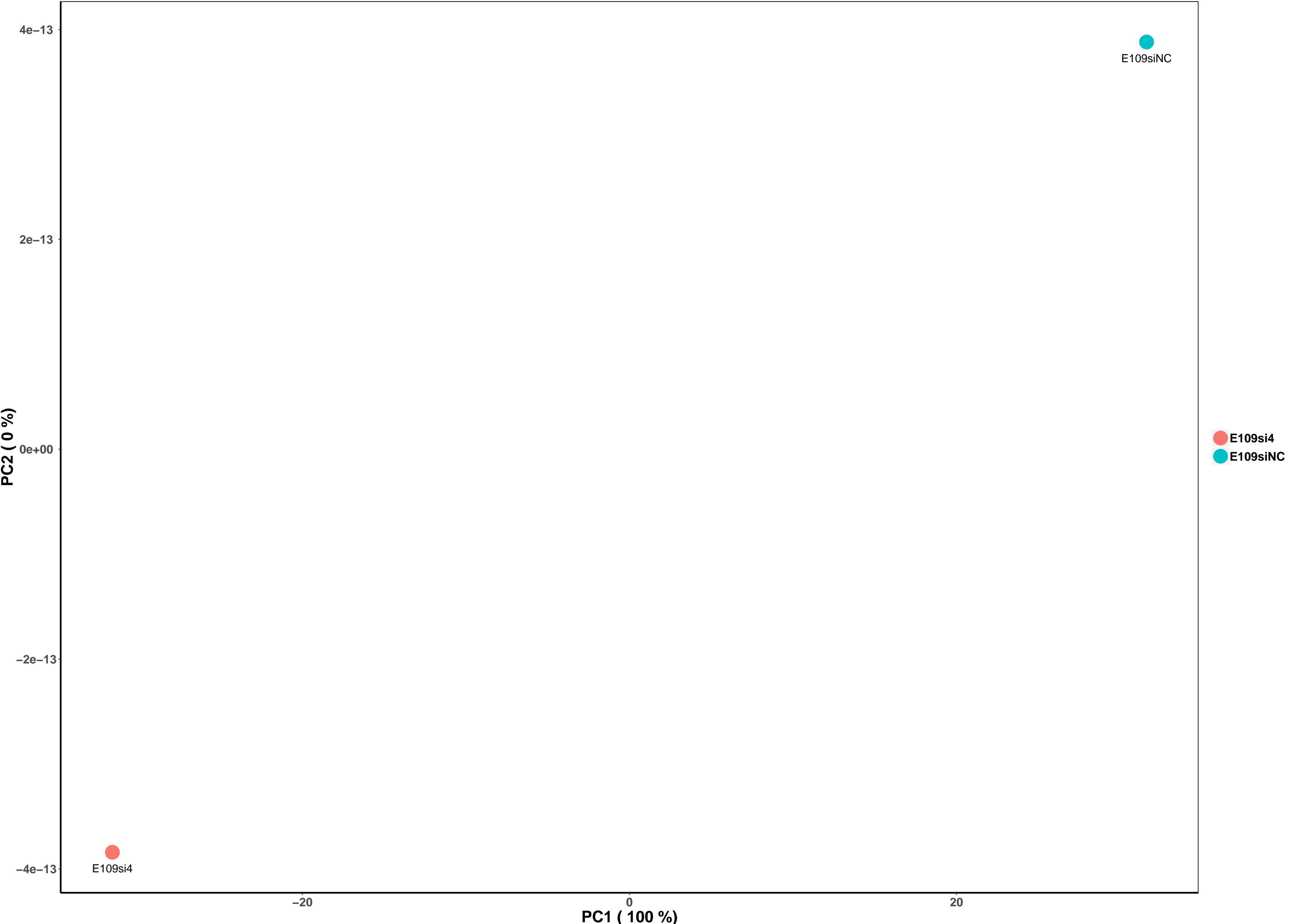

Supplement: Supplementary file 1 [file DataSheet1.zip › Results of eukaryotic transcriptome sequencing/genes/E109si4_vs_E109siNC/genes_PCA_score.pdf]

# E109si4\_vs\_E109siNC

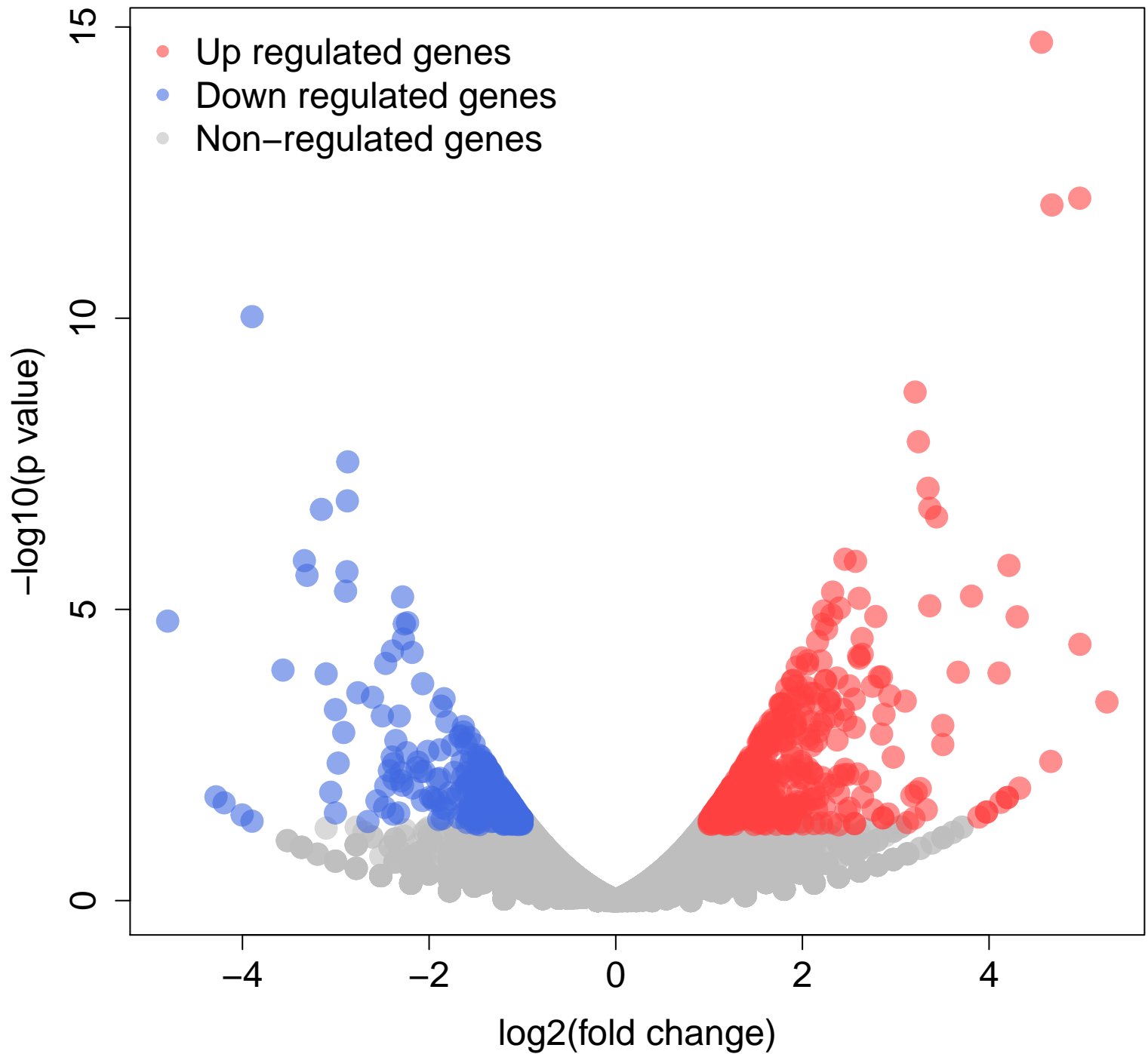

Supplement: Supplementary file 1 [file DataSheet1.zip › Results of eukaryotic transcriptome sequencing/genes/E109si4_vs_E109siNC/valcano.pdf]

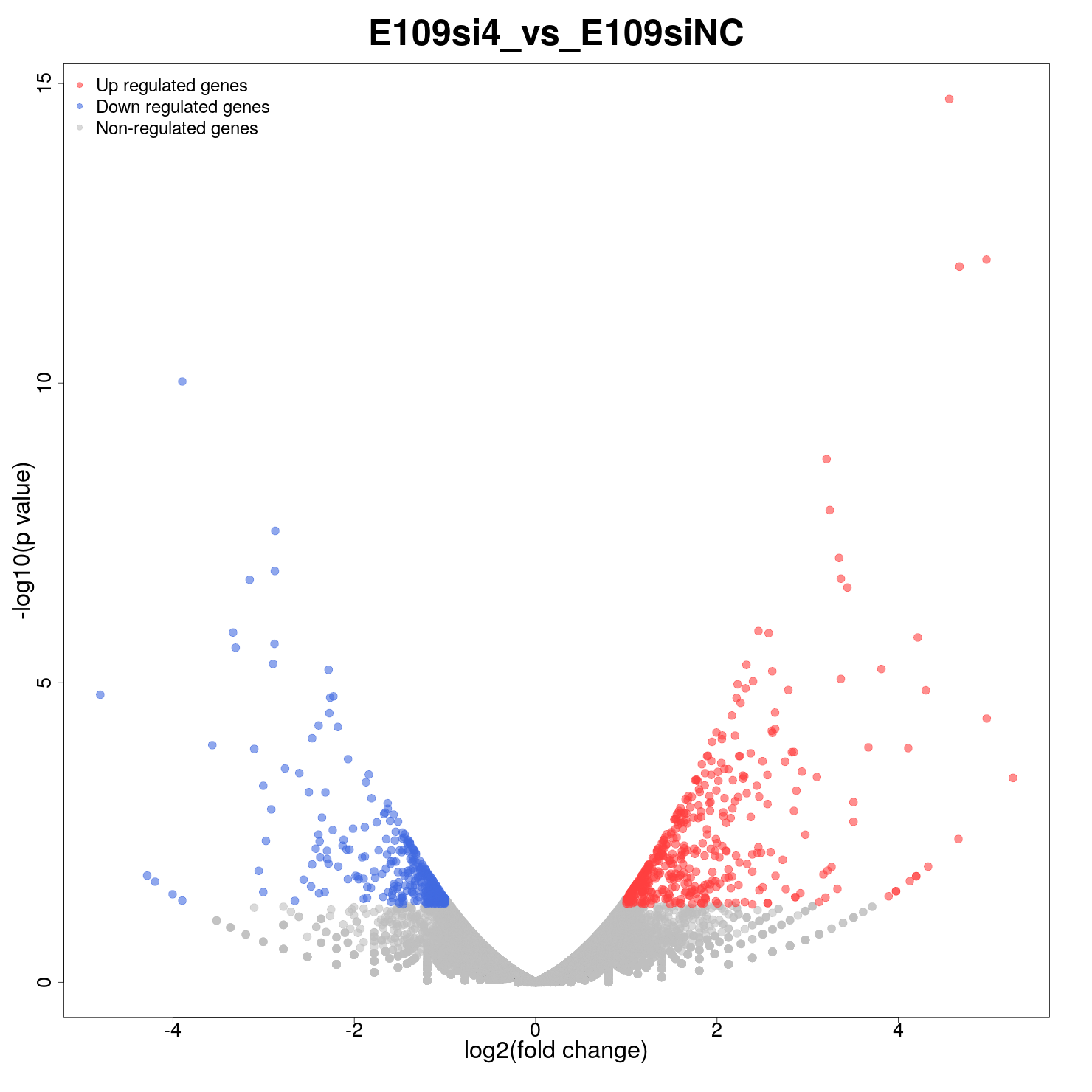

Supplement: Supplementary file 1 [file DataSheet1.zip › Results of eukaryotic transcriptome sequencing/genes/E109si4_vs_E109siNC/valcano.png]

PC score distribution

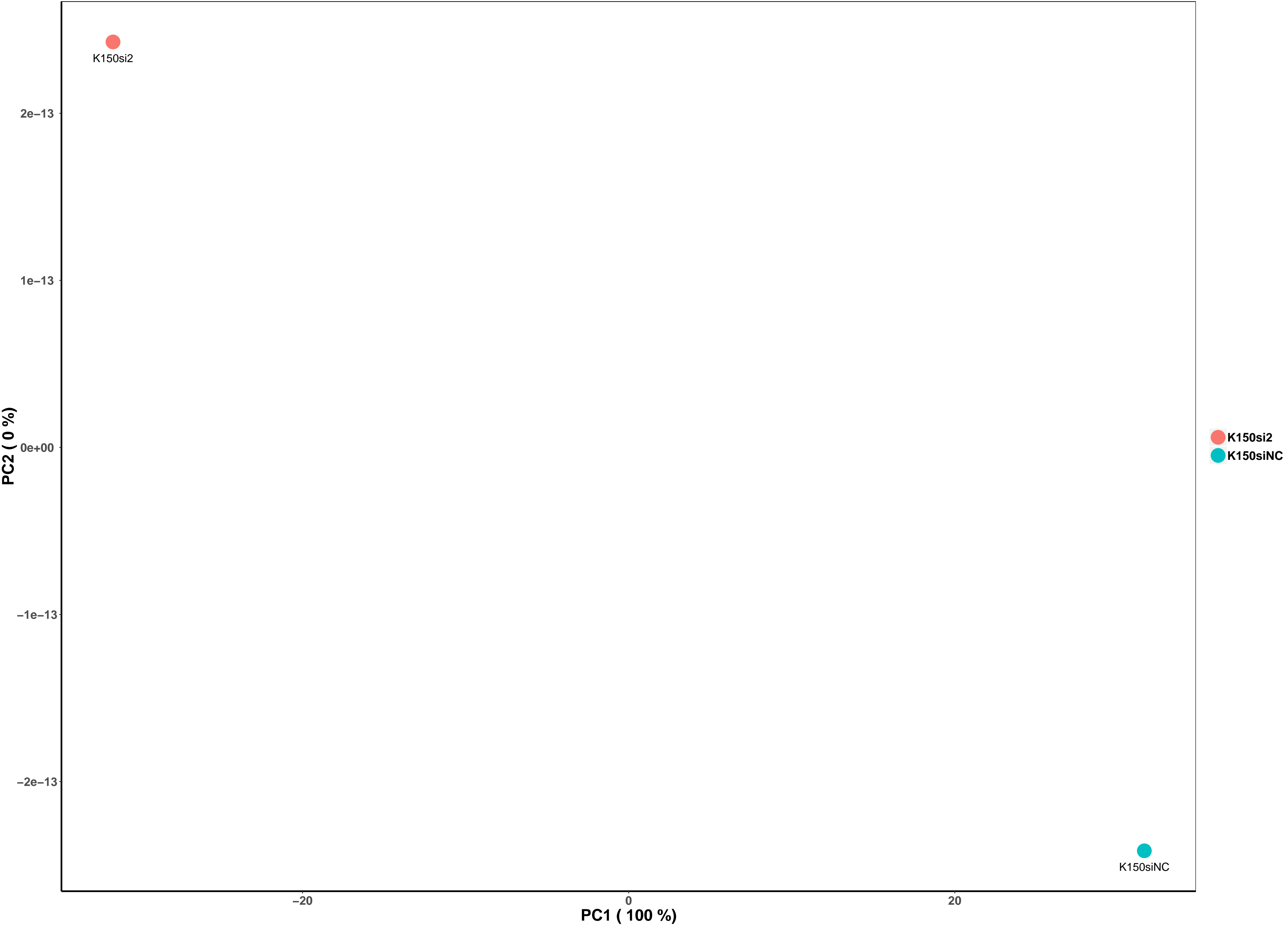

Supplement: Supplementary file 1 [file DataSheet1.zip › Results of eukaryotic transcriptome sequencing/genes/K150si2_vs_K150siNC/genes_PCA_score.pdf]

# K150si2\_vs\_K150siNC

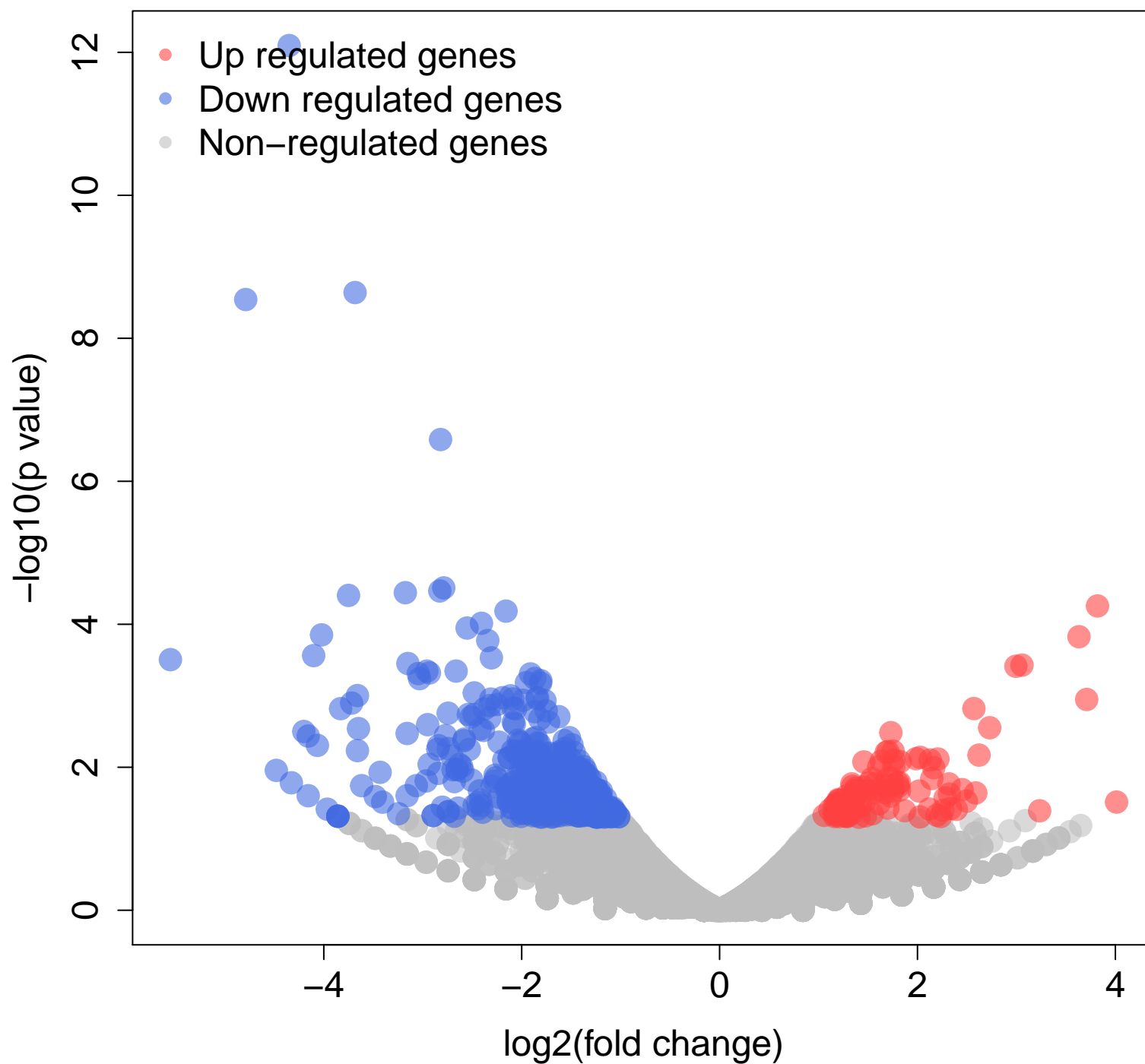

Supplement: Supplementary file 1 [file DataSheet1.zip › Results of eukaryotic transcriptome sequencing/genes/K150si2_vs_K150siNC/valcano.pdf]

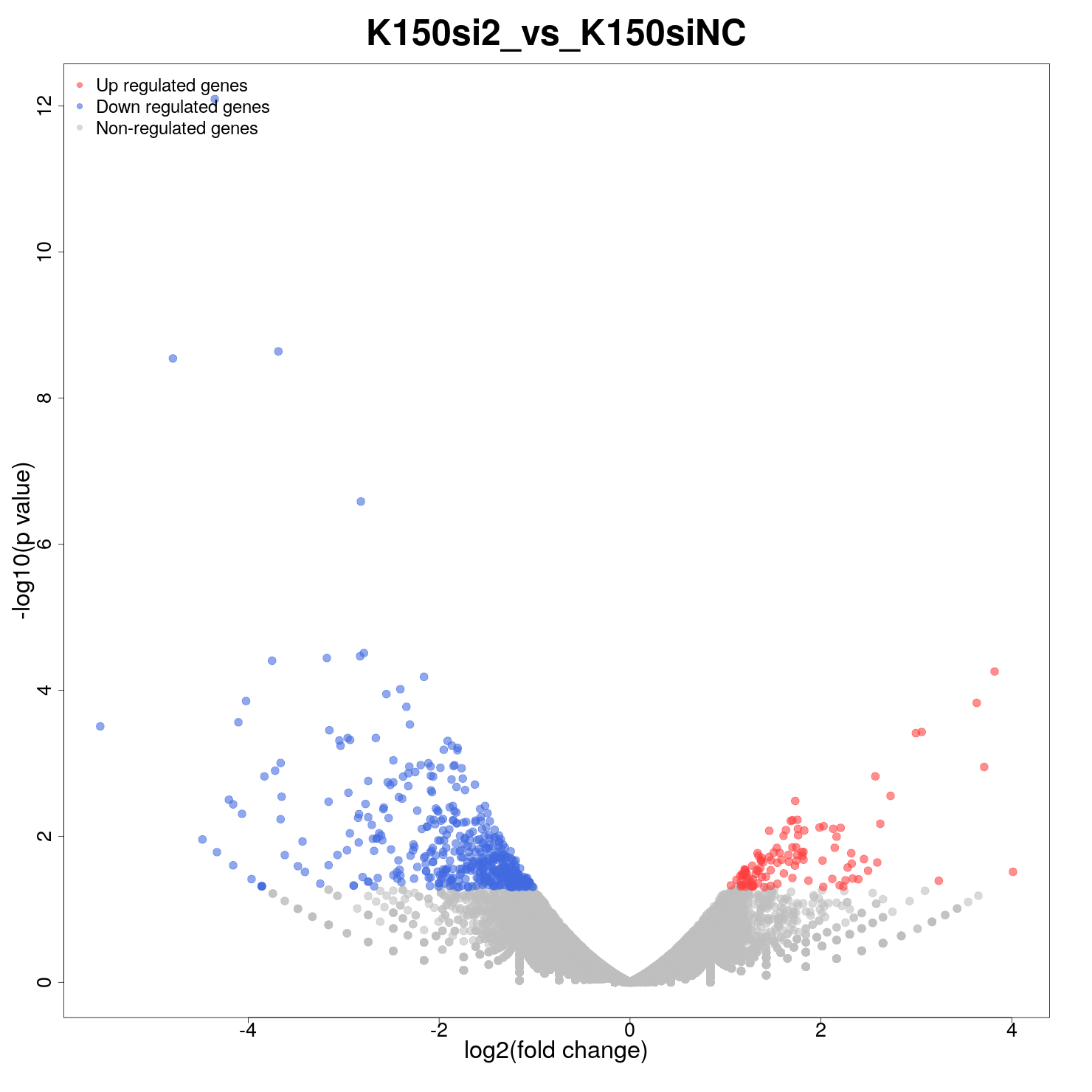

Supplement: Supplementary file 1 [file DataSheet1.zip › Results of eukaryotic transcriptome sequencing/genes/K150si2_vs_K150siNC/valcano.png]

PC score distribution

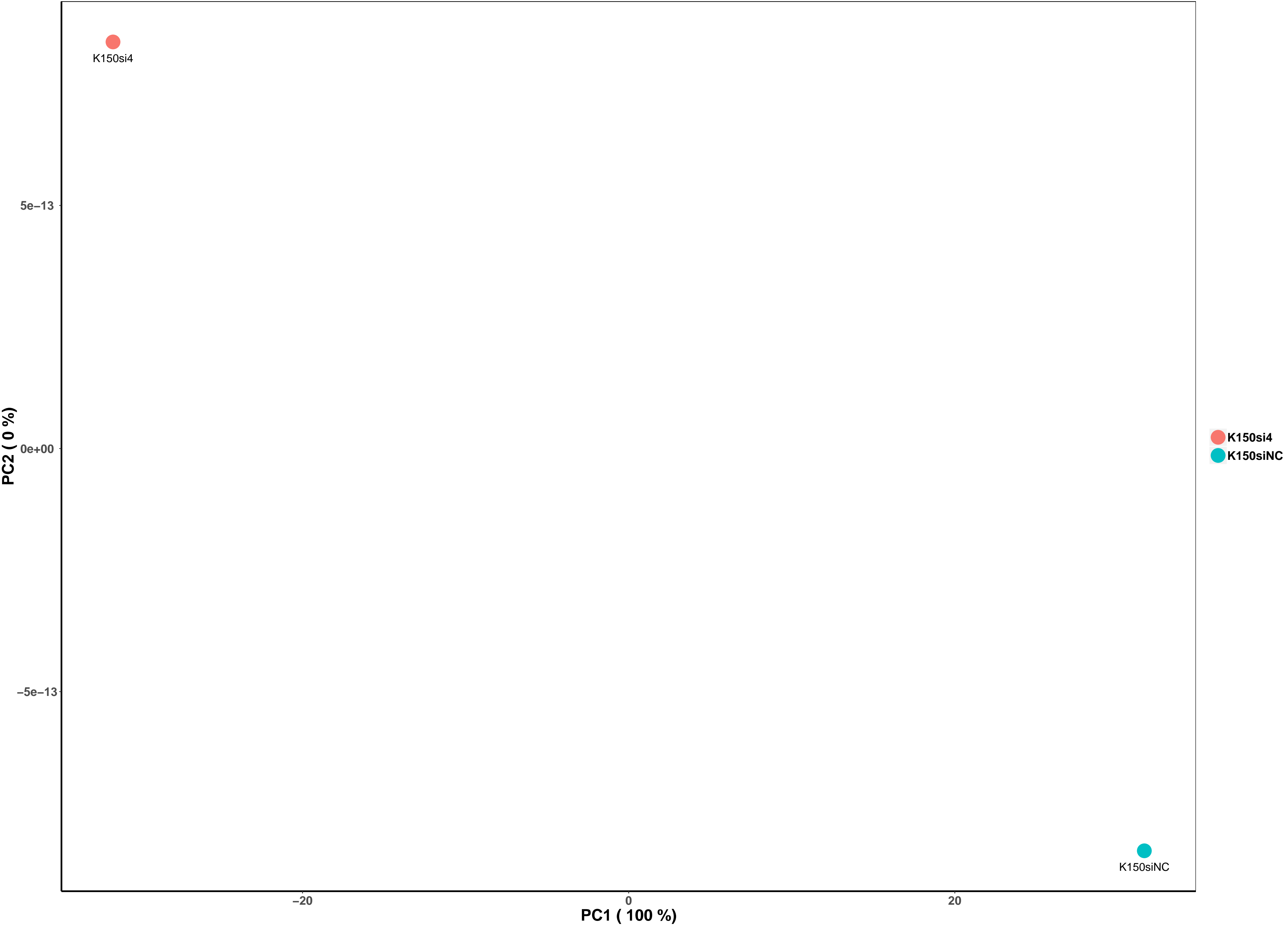

Supplement: Supplementary file 1 [file DataSheet1.zip › Results of eukaryotic transcriptome sequencing/genes/K150si4_vs_K150siNC/genes_PCA_score.pdf]

# K150si4\_vs\_K150siNC

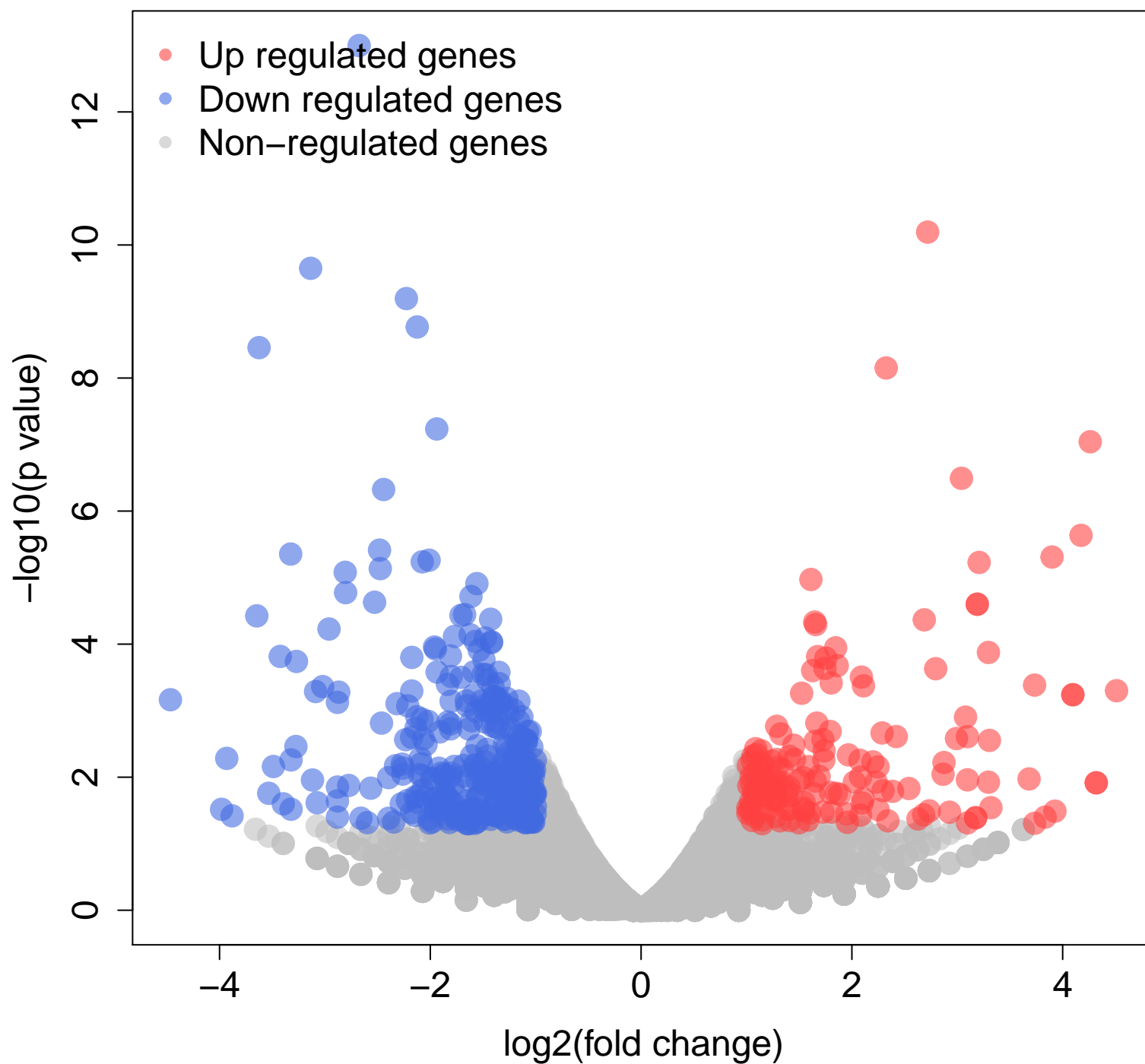

Supplement: Supplementary file 1 [file DataSheet1.zip › Results of eukaryotic transcriptome sequencing/genes/K150si4_vs_K150siNC/valcano.pdf]

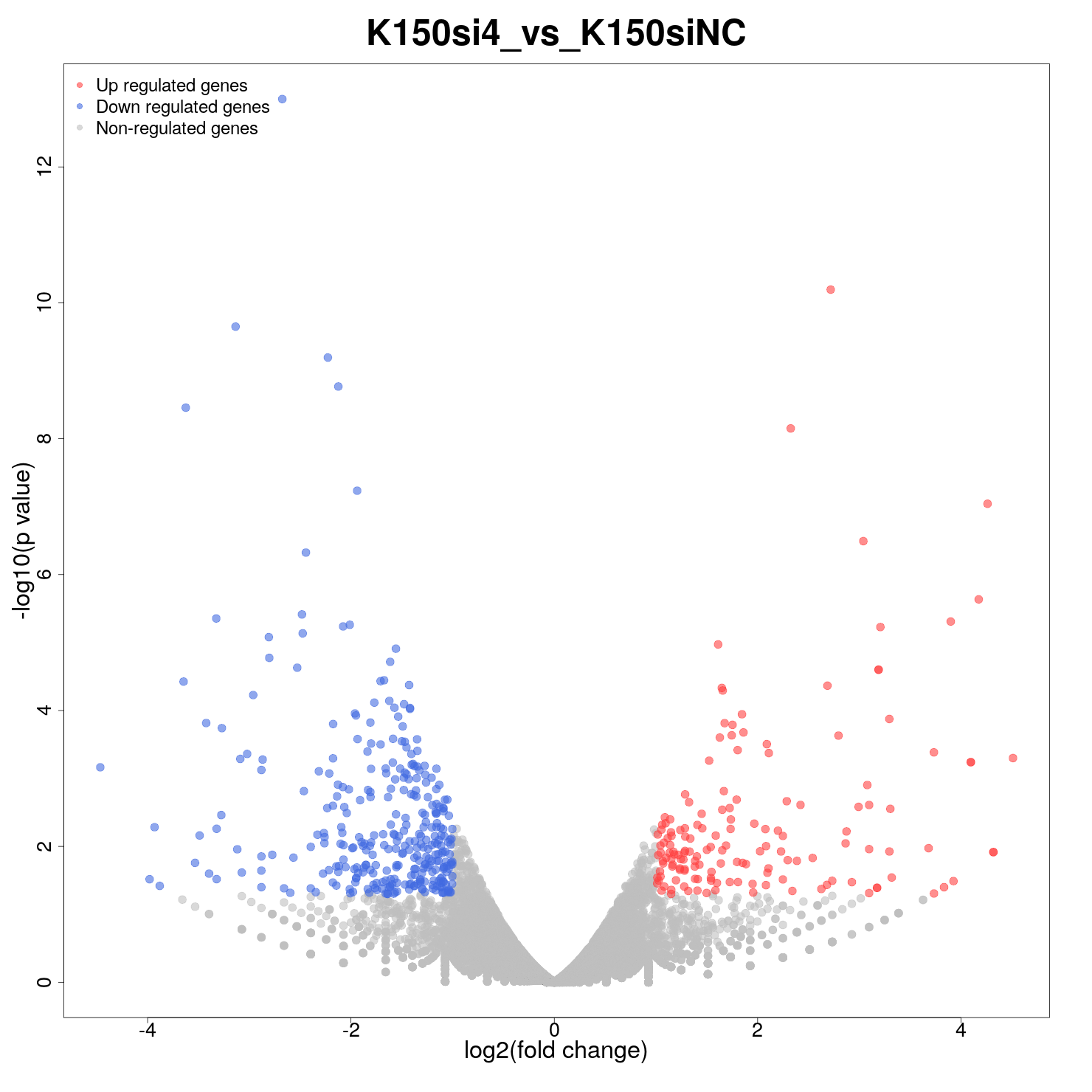

Supplement: Supplementary file 1 [file DataSheet1.zip › Results of eukaryotic transcriptome sequencing/genes/K150si4_vs_K150siNC/valcano.png]
