# Supplementary material for: Zinc finger protein ZC3H18 is abnormally expressed in esophageal cancer tissues and facilitates the proliferation of esophageal cancer cells
Source: Front Immunol. 2025 Feb 25;16:1556509. doi: 10.3389/fimmu.2025.1556509 (PMC11894379; doi:10.3389/fimmu.2025.1556509)
Supplement: Supplementary file 2 [file Table1.docx]

Supplement table 1：

General data of 25 patients with esophageal cancer

| Clinical and pathological features | N=25 | Percentage（%） |
| --- | --- | --- |
| Age |  |  |
| ≤60 | 8 | 32.0 |
| ＞60 | 17 | 68.0 |
| Sex |  |  |
| Male | 15 | 60.0 |
| Female | 10 | 40.0 |
| Histology  Squamous cell carcinoma  Adenocarcinoma  Tumor area  TOP  MID  BOT  TNM stage  I  II  III | 25  0  3  15  7  3  13  9 | 100.0  0.0  12.0  60.0  28.0  12.0  52.0  36.0 |
